# Biochemical and crystallographic investigations into isonitrile formation by a nonheme iron-dependent oxidase/decarboxylase

**DOI:** 10.1074/jbc.RA120.015932

**Published:** 2021-01-07

**Authors:** Rohan Jonnalagadda, Antonio Del Rio Flores, Wenlong Cai, Rimsha Mehmood, Maanasa Narayanamoorthy, Chaoxiang Ren, Jan Paulo T. Zaragoza, Heather J. Kulik, Wenjun Zhang, Catherine L. Drennan

**Affiliations:** 1 Department of Biology, Massachusetts Institute of Technology, Cambridge, Massachusetts, USA; 2 Department of Chemical and Biomolecular Engineering, University of California Berkeley, Berkeley, California, USA; 3 Department of Chemistry, Massachusetts Institute of Technology, Cambridge, Massachusetts, USA; 4 Department of Chemical Engineering, Massachusetts Institute of Technology, Cambridge, Massachusetts, USA; 5 Department of Chemistry, University of California Berkeley, Berkeley, California, USA; 6 California Institute for Quantitative Biosciences, University of California Berkeley, Berkeley, California, USA; 7 Chan Zuckerberg Biohub, San Francisco, California, USA; 8 Howard Hughes Medical Institute, Massachusetts Institute of Technology, Cambridge, Massachusetts, USA

**Keywords:** mononuclear Fe(II) α-ketoglutarate dependent dioxygenase, structure function, crystallography, stoichiometry, molecular dynamics, enzyme mechanism, CABA, (*R*)-3-((carboxylmethyl)amino)butanoic acid, INBA, (R)-3-isocyano butanoic acid, INLP, isonitrile lipopeptide, LC-HRMS, liquid chromatography–high-resolution mass spectrometry, RESP, restrained electrostatic potential

## Abstract

The isonitrile moiety is found in marine sponges and some microbes, where it plays a role in processes such as virulence and metal acquisition. Until recently only one route was known for isonitrile biosynthesis, a condensation reaction that brings together a nitrogen atom of l-Trp/l-Tyr with a carbon atom from ribulose-5-phosphate. With the discovery of ScoE, a mononuclear Fe(II) α-ketoglutarate-dependent dioxygenase from *Streptomyces coeruleorubidus*, a second route was identified. ScoE forms isonitrile from a glycine adduct, with both the nitrogen and carbon atoms coming from the same glycyl moiety. This reaction is part of the nonribosomal biosynthetic pathway of isonitrile lipopeptides. Here, we present structural, biochemical, and computational investigations of the mechanism of isonitrile formation by ScoE, an unprecedented reaction in the mononuclear Fe(II) α-ketoglutarate-dependent dioxygenase superfamily. The stoichiometry of this enzymatic reaction is measured, and multiple high-resolution (1.45–1.96 Å resolution) crystal structures of Fe(II)-bound ScoE are presented, providing insight into the binding of substrate, (*R*)-3-((carboxylmethyl)amino)butanoic acid (CABA), cosubstrate α-ketoglutarate, and an Fe(IV)=O mimic oxovanadium. Comparison to a previously published crystal structure of ScoE suggests that ScoE has an “inducible” α-ketoglutarate binding site, in which two residues arginine-157 and histidine-299 move by approximately 10 Å from the surface of the protein into the active site to create a transient α-ketoglutarate binding pocket. Together, data from structural analyses, site-directed mutagenesis, and computation provide insight into the mode of α-ketoglutarate binding, the mechanism of isonitrile formation, and how the structure of ScoE has been adapted to perform this unusual chemical reaction.

Isonitrile, an electron-rich functional group, is a hallmark of a variety of natural products such as xanthocillin and rhabduscin and plays a role in diverse processes such as metal acquisition and virulence (Fig. S1) (1). The biosynthesis of the isonitrile group was thought to be restricted to the isonitrile synthase (IsnA) enzyme family. These enzymes catalyze the formation of isonitriles using the nitrogen atom of an α-amino group of l-Trp/l-Tyr and a carbon atom from ribulose-5-phosphate (Fig. S1) (2). Recent studies of a conserved gene cluster in some Actinobacteria species, including *M. tuberculosis*, revealed a novel biosynthetic pathway for isonitrile synthesis as part of the formation of nonribosomally synthesized isonitrile lipopeptide (INLP). Furthermore, a study of the gene cluster from *Streptomyces coeruleorubidus* showed that isonitrile biosynthesis is catalyzed by the enzyme ScoE (3). Notably, unlike the IsnA enzymes, ScoE forms an isonitrile moiety from a single substrate: a β-glycine adduct attached to a short fatty acyl chain, as confirmed by isotope labeling (3) (Fig. 1). ScoE therefore represents a novel enzymatic mechanism of isonitrile biosynthesis (3).

**Figure 1:**
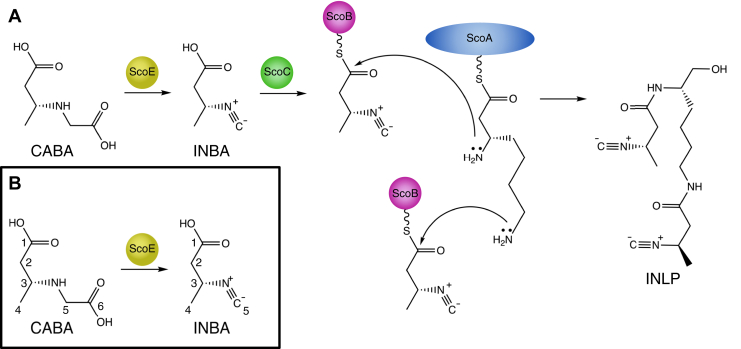
**Reaction catalyzed by ScoE.***A*, ScoE is part of a nonribosomal peptide biosynthetic pathway that makes an isonitrile lipopeptide (INLP) and acts on an untethered substrate. *B*, isotope labeling studies have confirmed that the carbon–nitrogen single bond in CABA is converted to the isonitrile moiety. The full four-electron oxidation is catalyzed by the Fe(II)/αKG dioxygenase ScoE. The carbon numbering is indicated.

Bioinformatic analysis of the INLP-producing gene clusters suggested that the *scoE* gene encodes a nonheme iron(II) α-ketoglutarate (α-KG)-dependent dioxygenase (Fe(II)/αKG-dioxygenase) (3). This prediction was confirmed when it was shown that both iron(II) and α-KG were required by ScoE *in vitro* for isonitrile formation on an untethered substrate, (R)-3-carboxyaminobutanoic acid (CABA), to form an untethered product, (R)-3-isocyano butanoic acid (INBA) (Fig. 1) (4). This finding firmly places ScoE in the Fe(II)/αKG-dioxygenase superfamily, a family of enzymes that uses a mononuclear Fe(II) cofactor, α-KG, and molecular oxygen to catalyze a diverse set of reactions, such as hydroxylation, halogenation, carbon–carbon bond desaturation, and ring contraction (5). Notably, isonitrile formation is a new activity in this enzyme family.

Independent of the particular reaction, all Fe(II)/αKG-dioxygenases are thought to share a common mechanism of activation (Fig. S2) (5). This activation chemistry occurs at the mononuclear Fe(II) cofactor, which is coordinated by a conserved 2-histidine-1-carboxylate facial triad motif (2-His-1-Asp for ScoE) that acts as three ligands to the metal. The cosubstrate α-KG binds the metallocofactor in a bidentate fashion, leaving one open coordination site, usually found in close proximity to the substrate binding site (6). This final coordination site is the binding site of molecular oxygen, at which point chemistry begins. Specifically, molecular oxygen is cleaved, with one oxygen atom inserting into α-KG to form succinate and carbon dioxide, and the other atom remaining bound to the metallocofactor to form a highly reactive Fe(IV)=O species (7, 8, 9) (Fig. S2). This reaction step is thought to occur primarily in the presence of substrate, but it has been observed in the absence of substrate as well, generally at a lower rate (uncoupled reaction) (10). The Fe(IV)=O species performs a hydrogen atom abstraction from the substrate to form an Fe(III)-OH species and a substrate radical (Fig. S2) (11). The resolution of this substrate radical differs between enzymes of this family. The best understood reaction of this enzyme family is hydroxylation, in which the Fe(III)-OH bond is homolytically cleaved such that the hydroxyl group “rebounds” to the substrate radical to yield a hydroxylated product and restore the Fe(II) cofactor (Fig. S3) (12). The hydroxylase TauD is the most studied Fe(II)/αKG-dioxygenase and is considered to be the archetypal enzyme of the family (5).

Inspection of the reaction catalyzed by ScoE (Fig. 1) has led to the proposal that like TauD, ScoE catalyzes hydroxylation reactions as part of the four-electron oxidation of CABA (Fig. 2) (4, 13). The sites of the hydroxylation(s) are unknown, but the α-carbon of the glycyl moiety has been proposed to be both singly and doubly hydroxylated, and the nitrogen of the glycyl moiety has also been proposed to be a site of hydroxylation (Fig. 2). Chang and colleagues proposed a C5-hydroxylated CABA compound as an intermediate in the reaction from ScoE’s homologue, SfaA, from *Streptomyces thioluteus* based on a ^13^C-NMR spectrum obtained by using a 5-^13^C-CABA substrate (13). However, this putative intermediate was not chemically synthesized and tested *in vitro* to determine whether it can be directly converted to INBA by ScoE. Regardless of the site(s) of hydroxylation, these mechanistic proposals would require two equivalents of α-KG and two equivalents of molecular oxygen. Chang and colleagues found that 4.5 equivalents of succinate were produced for every equivalent of isonitrile product generated by ScoE and suggested that this unexpectedly high succinate-to-isonitrile ratio could be due to uncoupling of α-KG cleavage from INBA production (13). Here, we investigate the stoichiometry of this reaction and confirm that one CABA is consumed and one INBA is generated per two equivalents each of α-KG and molecular oxygen. Two equivalents of succinate are generated.

**Figure 2:**
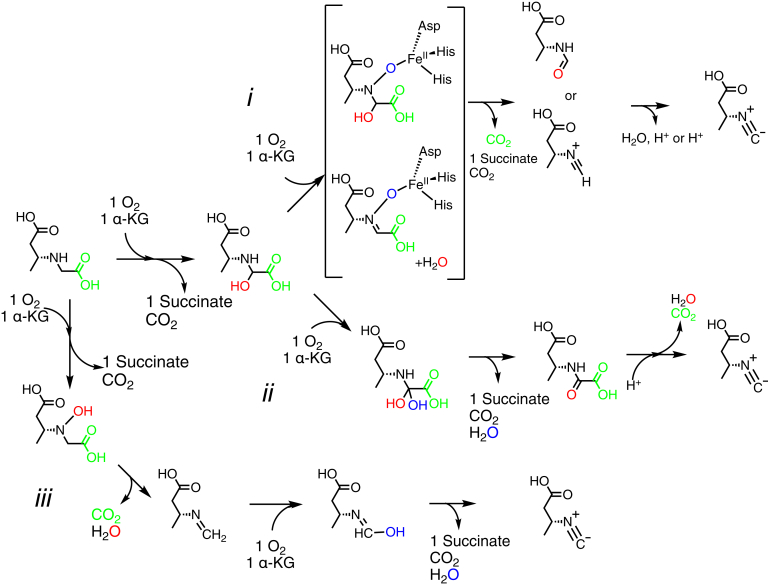
**Possible mechanisms of isonitrile formation by ScoE.** Top schemes (*i* and *ii*) show αKG-dependent hydroxylation at C5 of CABA occurring first. Schemes *i* and *ii* differ in the second half-reaction with αKG. These schemes are based on reaction mechanisms proposed by Chang and coworkers (13). In scheme *iii*, αKG-dependent hydroxylation of N occurs before αKG-dependent hydroxylation of C5 of CABA.

Structures of ScoE have also been determined. The first crystal structure of ScoE revealed the archetypal fold containing a double-stranded β-helix fold (also known as the jelly roll fold), showing that it is consistent with this family of enzymes (4). The structure also showed the expected 2-His-1-Asp motif for Fe(II) binding (4). However, this structure lacked physiological ligands and the physiological metal, with Zn(II), choline, and acetate observed instead of Fe(II), CABA, and α-KG, respectively (4). Another study reported a crystal structure of ScoE bound to Fe(II), CABA, and the α-KG analog tartrate at 2.18 Å resolution (13). Here, we present a higher-resolution structure of ScoE with Fe(II) and CABA bound at 1.7 Å resolution and evaluate its binding interactions with the protein through mutagenesis. We go on to solve crystal structures of ScoE with α-KG bound in an unexpected conformation that impinges on the CABA binding site. We use structural comparisons between the ScoE structures to identify a conformational change that repositions the residues Arg157 and His299 by approximately 10 Å in the active site, creating what appears to be an inducible binding site for α-KG that does not impinge on the CABA binding site. Together with a crystal structure of ScoE bound with oxovanadium, a stable mimic of the Fe(IV)-oxo intermediate, we consider how these structural, biochemical, and computational data inform our understanding of this unprecedented reaction for this enzyme superfamily.

## Results

### The stoichiometry of the ScoE reaction was determined

Metal-free ScoE of *S. coeruleorubidus* was purified heterologously from *Escherichia coli*, with the Fe(II) cofactor reconstituted *in vitro* and used to determine reaction stoichiometry. Product INBA formation was indirectly detected and quantified using 3,6-di-2-pyridyl-1,2,4,5-tetrazine (Py-tetrazine), which reacts with INBA to generate 3,5-di(pyridine-2-yl)-1H-pyrazol-4-amine (Py-aminopyrazole) (14). No free INBA was detected after the tetrazine click reaction (data not shown), indicating complete conversion of INBA to Py-aminopyrazole. Oxygen consumption was quantified using an Oxygraph Hansatech probe at room temperature (Fig. S4). Enzymatic reactions were incubated until the oxygen signal plateaued, indicating the completion of the reaction. Part of the assay mixture was subsequently quenched with cold MeOH, and the remaining mixture was quenched with cold MeOH supplemented with Py-tetrazine to obtain the enzymatic stoichiometry using liquid chromatography–high-resolution mass spectrometry (LC-HRMS). Standards containing varying concentrations of α-KG, succinate, CABA, and Py-aminopyrazole, dissolved in 50 mM HEPES at pH 8.0, were prepared to quantify their relative concentrations in the enzymatic assays (Fig. S5).

The results from these stoichiometry quantification experiments show the expected 1:1:1 correlation between α-KG consumption, oxygen consumption, and succinate formation (Table 1). CABA consumption and INBA production are also 1:1 as expected. The stoichiometry between α-KG consumed/succinate produced and CABA consumed/INBA produced is 2:1 (summarized in Table 1). A previous study reported a 4.5:1 stoichiometry between succinate and INBA, which as the authors’ suggested was artifactually high in succinate, most likely due to unproductive cleavage of α-KG (13). However, in the absence of other measurements of products formed and reactants consumed, the degree of α-KG reaction uncoupling was unknown, and it was impossible to determine whether 4.5:1 stoichiometry was actually 2:1 or 1:1. In this study, the complete quantification of reactants and products involved in the ScoE enzymatic reaction has been determined, showing that two equivalents of α-KG are necessary for INBA production, indicating that two α-KG-dependent half-reactions must be involved in isonitrile biosynthesis.

**Table 1:** Observed consumption of ScoE reactants and generation of ScoE products

Reaction	CABA consumption (μM)	α-KG consumption (μM)	Oxygen consumption (μM)	Succinate production (μM)	INBA production (μM)
100 μM ScoE Assay with 90 μM (NH_4_)_2_Fe(SO_4_)_2_	108 ± 3.3	221.4 ± 28.4	221.5 ± 13.5	230.1 ± 15.1	116.4 ± 15.2

Concentrations were obtained from the LC-HRMS and Oxygraph Hansatech Probe experiments for the ScoE reaction. Each parameter listed above was quantified from the same reaction and was verified in triplicate. The values and uncertainty correspond to the mean and standard deviation, respectively.

### α-KG can react with O_2_ in the absence of CABA

With a stoichiometry of two α-KG consumed for each CABA consumed, we next considered whether the first α-KG dependent reaction could occur prior to CABA binding. Although typically in this enzyme superfamily substrate and α-KG both bind before the reaction of α-KG with O_2_ begins (5), there is precedent for α-KG binding and the generation of an off-line Fe(IV)=O species prior to substrate binding (15,16). In the latter case, substrate binding is thought to cause the off-line Fe(IV)=O species to reposition to an online configuration that is appropriate for hydrogen atom abstraction (17).

We tested whether CABA binding is required for the consumption of oxygen and α-KG by measuring oxygen consumption in the presence and absence of CABA. As a control, we measured the consumption of oxygen in the absence of α-KG, and under this condition, no oxygen consumption was observed (Fig. S6). Following this control experiment, we observed clear consumption of oxygen by ScoE in the presence of α-KG and in the absence of CABA, with a possible plateau in the oxygen consumption after approximately 300 s. Addition of CABA results in further oxygen consumption, and the rate of this oxygen consumption appears to be greater when CABA is present (Fig. S6). Thus, although ScoE can react with α-KG in the absence of CABA, we did not measure the rate of this reaction in this work.

### CO_2_ is a reaction product of ScoE reaction with CABA

The carbon and two oxygen atoms of the carboxylate group of CABA are lost in the formation of INBA (Fig. 1), but no experimental evidence has been previously reported to establish whether this group is lost as a result of decarboxylation, decarbonylation, or deformylation (4,13). To address this question, [6-^13^C]-CABA was chemically synthesized and used as a substrate for an *in vitro* ScoE assay, where the presence of [^13^C]-CO_2_ or [^13^C]-CO was monitored using gas chromatography-mass spectrometry (GC-MS). The release of [^13^C]-CO_2_ as a product of the ScoE catalyzed INBA formation was confirmed (Fig. S7). In contrast, [^13^C]-CO was not detected in the GC-MS experiments nor was CO detected spectroscopically using reduced myoglobin, which exhibits a Soret shift when CO binds to the myoglobin heme (Fig. S8) (18). The release of [^13^C]-CO_2_ was further detected from an *in vitro* biochemical assay containing commercially purchased 1,2,3,4-^13^C α-KG and unlabeled CABA using GC-MS (Fig. S6). The [^13^C]-CO_2_ peak area ratio between the 1,2,3,4-^13^C α-KG and [6-^13^C]-CABA experiments was 2:1 and in corroboration with the reaction stoichiometry determined here. To test possible deformylation of CABA, two independent analytical assays were performed and both experiments yielded a negative result, failing to detect formate in our ScoE reaction. Specifically, a Sigma-Aldrich Formate Assay kit failed to detect formate; and no formate adduct was detected through LC-HRMS following a click reaction with 2-nitrophenylhydrazine (Fig. S9) (18).

### H_2_O_2_ is not produced by ScoE, indicating that water is the likely product of O_2_ reduction

We additionally investigated whether O_2_ is reduced to hydrogen peroxide (H_2_O_2_) as a result of the ScoE-catalyzed reaction. Two independent analytical assays were used to test for the presence of H_2_O_2_. First, a commercial Amplex Red H_2_O_2_ Detection kit was used. Second, production of H_2_O_2_ by ScoE was probed through a coupled assay with the enzyme catalase. Catalase converts H_2_O_2_ to O_2_, the production of which can be monitored using an Oxygraph Hansatech according to a previously described method (19). Both sensitive analytical methods yielded a negative result for H_2_O_2,_ production, indicating that oxygen is likely reduced to water.

### CABA binds to ScoE in a similar orientation to taurine in TauD

**Figure 3:**
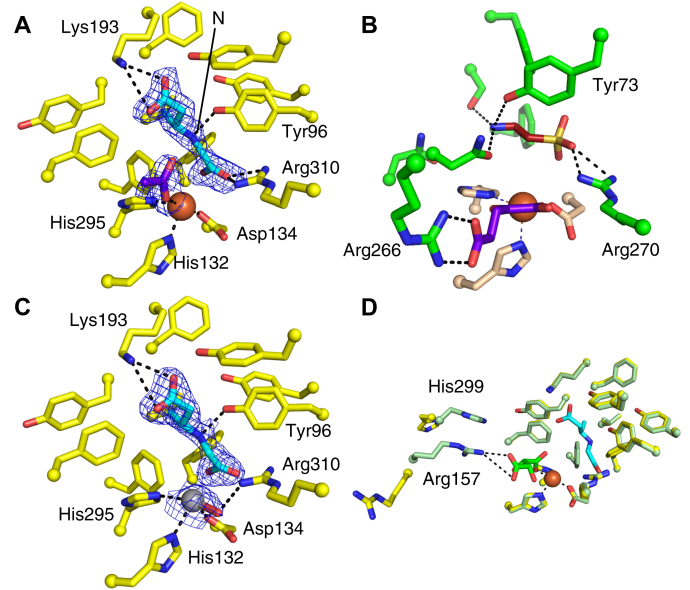
**Active site comparisons.***A*, ScoE with CABA (*cyan*), Fe(II) (*orange sphere*), and acetate (*purple*). For clarity, the nitrogen atom of CABA is labeled. *B*, TauD (1OS7) with taurine (*red*), α-KG (*purple*), Fe(II) (*orange sphere*), and facial triad (wheat). *C*, ScoE with CABA (*cyan*) and oxovanadium (*gray* with oxygen atom in *red*). *D*, superposition of ScoE structures shows two positions of Arg157 and His299. The structures solved in this work are shown in *yellow*, the previously determined structure (with tartrate bound, PDB: 6L6X) is shown in *green*. CABA is shown in *cyan*, tartrate in *purple*, and Fe(II) as an *orange sphere*. Maps shown in *blue mesh* above are *2F_o_−F_c_* composite omit maps contoured at 1 σ. *Dashed lines* indicate close interactions (less than 4.0 Å).

To probe the structural basis of isonitrile formation by an Fe(II)/αKG-dioxygenase, a structure of ScoE from *S. coeruleorubidus* was solved to 1.70 Å resolution with CABA bound (Table S1). In the ScoE active site, clear electron density was observed for a bound CABA (Fig. 3*A*) in the same location that was proposed to be the CABA binding site from the choline-bound ScoE structure (4) and shown to be the CABA binding site in the recent ScoE structure (13). As expected (4, 13), Fe(II) is bound at the conserved 2-His-1-Asp facial triad motif. An acetate molecule from the crystallization buffer occupies the putative α-KG binding site. The CABA binding site in ScoE is reminiscent of the taurine binding site in the archetypal α-KG-dependent dioxygenase TauD from *E. coli* (Fig. 3*B*) (20). In both active sites, the respective substrate is bound axial to a histidine residue in the conserved facial triad. A conserved arginine (Arg310 in ScoE; Arg270 in TauD) coordinates the carboxylate moiety of CABA in ScoE and the sulfonate moiety of taurine in TauD. CABA is further locked in the active site by an interaction between the other substrate carboxylate moiety and Lys193.

### A conformational change in lysine 193 occurs upon CABA binding

**Figure 4:**
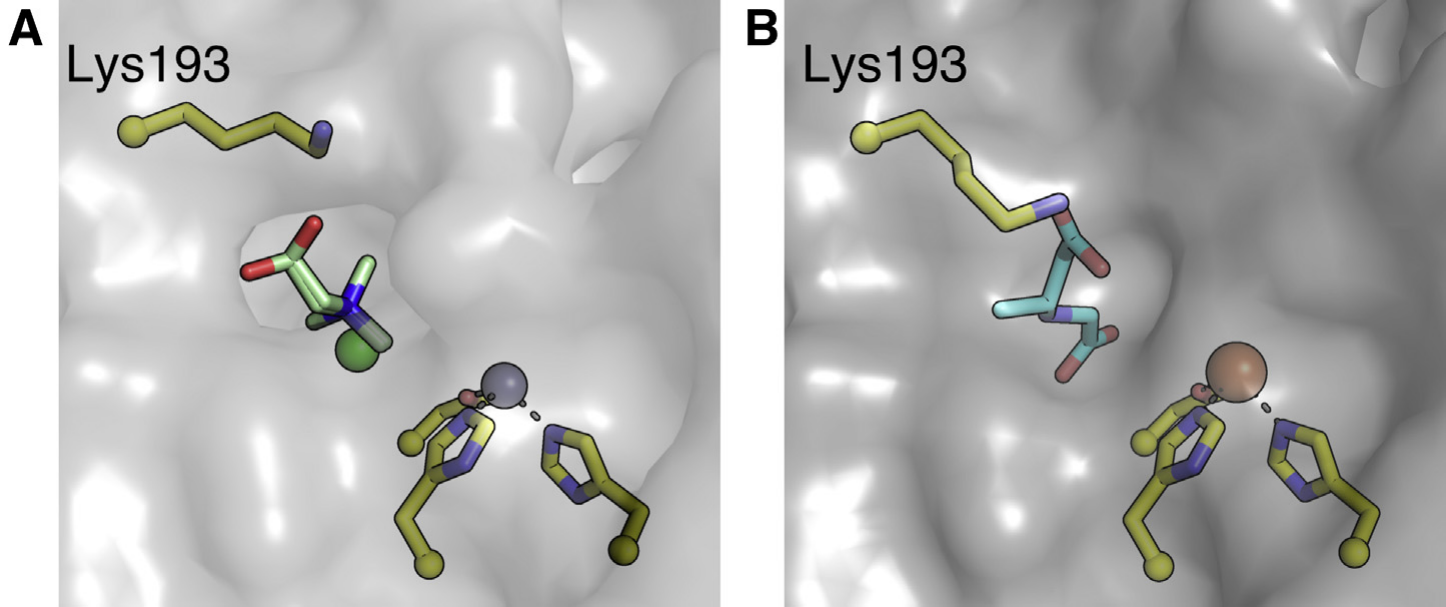
**Comparison of ScoE surface at Lys193 in the presence of choline and CABA.***A*, surface of ScoE protein with choline, chloride, and Zn(II) in the active site (PDB: 6DCH). Choline is shown in *green*, chloride is shown as a *green sphere*, and Zn(II) is shown as a *purple sphere*. The protein surface is represented in *gray*. Lys193 is labeled and is flipped out, exposing the active site to solvent. *B*, surface of ScoE protein with CABA and Fe(II) bound in the active site. CABA is shown in *cyan* and Fe(II) is shown as an *orange sphere*. The protein surface is represented in *gray*. Lys193 is flipped in to make a hydrogen bond with CABA and close the active site.

A comparison of the choline-bound and CABA-bound ScoE structures reveals a conformational change of Lys193 that positions the primary amine of the side chain to interact with CABA (Fig. 4). In the presence of nonsubstrate choline, the side chain of Lys193 is flipped away from the ScoE active site. As Lys193 is at the protein surface, the “flipped out” conformation of this side chain exposes the CABA binding site to solvent. When CABA binds, Lys193 flips in, burying the untethered CABA (4) in the active site (Fig. 4*B*).

### An ordered water network extends from the CABA binding site to the protein surface

**Figure 5:**
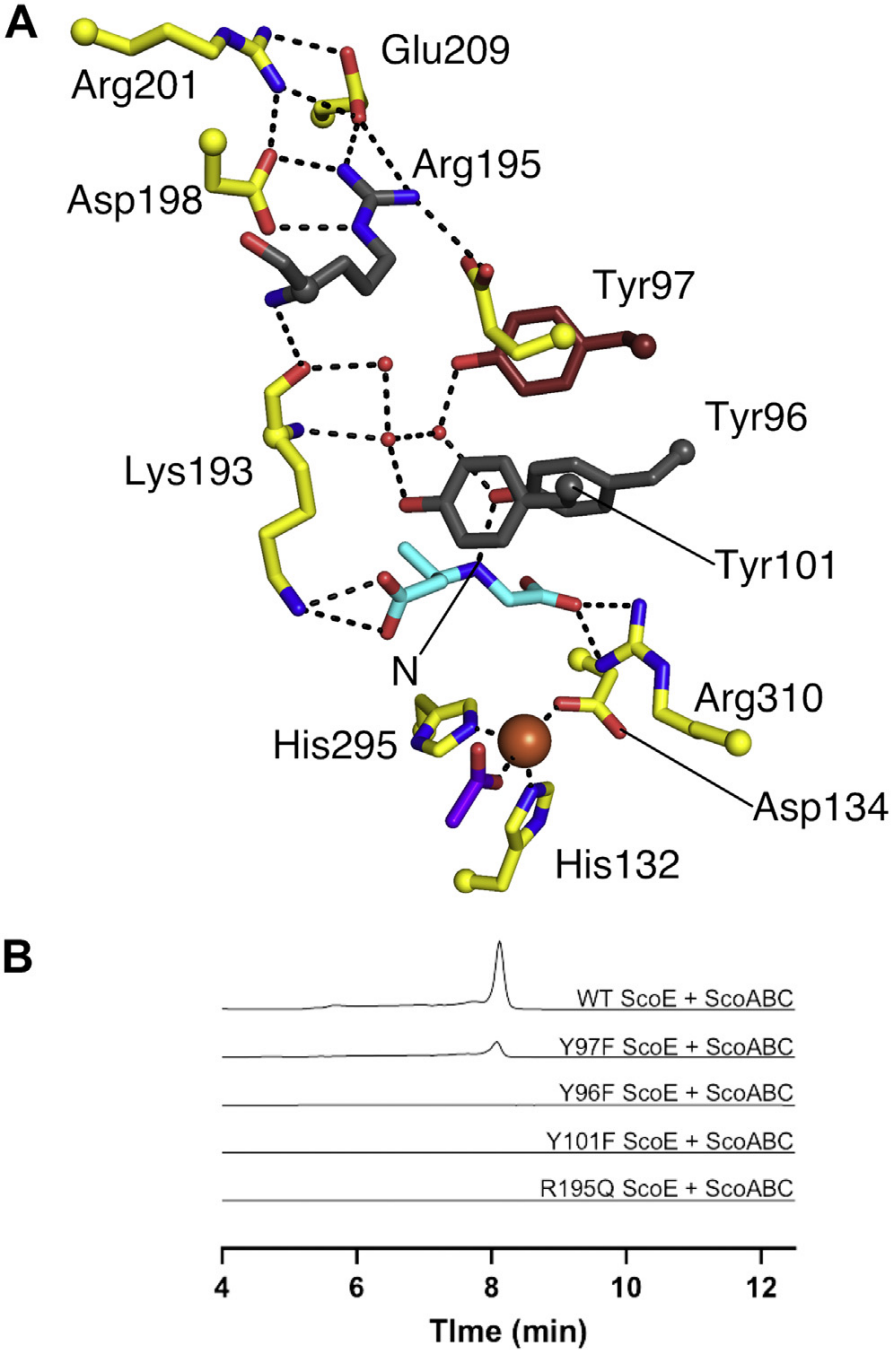
**Hydrogen bonding network in ScoE extends from CABA to the protein surface.***A*, CABA is shown in *cyan*, acetate is shown in *purple*, and Fe(II) is shown as an *orange sphere*. Tyr96, Tyr101, and Arg195 are shown in *gray*, indicating that product is not detected when these residues are substituted. Tyr97 is shown in red to indicate that substitution of this residue results in reduced product formation. *Dashed lines* indicate close interactions (less than 4.0 Å). For clarity, the nitrogen atom of CABA is labeled. *B*, extracted ion chromatograms corresponding to the production of the INLP (calculated:[M + H]+= 323.2078, observed:[M + H]+= 323.2093, 4.6 ppm error) from coupled ScoABCE assays using wild-type ScoE and four single amino acid variants. In all cases, the calculated masses with a 10-ppm error tolerance were used. A reaction mixture comprised of 50 mM HEPES, pH 8, 500 μM α-KG, 2 mM CABA, 100 μM Apo-ScoE, and 90 μM (NH_4_)_2_Fe(SO_4_)_2_ was gently mixed and incubated for 10 min at room temperature. After incubation, the reaction was mixed with 25 μl of 2 mM MgCl_2_, 5 mM ATP, 500 μM lysine, 4 mM NADPH, 50 μM ScoA, 50 μM ScoB, and 20 μM ScoC.

The observed orientation of CABA in the ScoE active site appears to be maintained by electrostatic interactions with Lys193 and Arg310 on either end of the CABA substrate and by a hydrogen bond between Tyr96 and the CABA secondary amine in the middle of the CABA molecule (Fig. 5). To test the importance of this interaction, we prepared a ScoE variant in which this tyrosine residue was substituted with phenylalanine (ScoE-Y96F) and detected no product formation from this variant in an *in vitro* assay (Fig. 5*B*). This protein:CABA interaction helps organize a larger hydrogen bonding network that extends to the protein surface. Tyr96, Tyr97, Tyr101, and the backbone of Lys193 form hydrogen bonds to stabilize three ordered water molecules within the protein (Fig. 5*A*). Furthermore, the backbone amine of Lys193 makes a hydrogen bond with the backbone carbonyl of Arg195, aiding in positioning this side chain for hydrogen bonding to the side chains of Asp198 and Glu209. Both of these side chains also make hydrogen bonds with Arg201 at the surface of the protein (Fig. 5*A*).

We examined the importance of this hydrogen bonding network by altering residues in this network (ScoE-Y101F and ScoE-R195Q) (Fig. 5*A*). Product formation was not detected from *in vitro* ScoE activity assays with either variant (Fig. 5*B*). Product formation was however detected *in vitro* with another variant, ScoE-Y97F, but the yield of product appears to be lower than that of wild-type ScoE, suggesting that the hydrogen bonding network is compromised but still intact in this variant. Strikingly, the observed loss of activity in both ScoE-Y101F and ScoE-R195Q variants despite the preservation of the hydrogen bond at Tyr96 suggests that the hydrogen bonding network plays a catalytic role in isonitrile formation assuming that the structural integrity of the protein is maintained.

### A ScoE structure with a Fe(IV)-oxo mimic demonstrates a potential off-line oxo species

To probe the structural basis for the reaction of CABA with Fe(IV)=O, we used vanadium(IV), which in the presence of oxygen has been demonstrated to be a suitable and stable mimic of the Fe(IV)=O intermediate for use in X-ray crystallography (17, 21, 22). Crystals of ScoE with oxovanadium were obtained by aerobic crystallization of ScoE in the presence of vanadium(IV) sulfate and CABA, and the structure was solved to 2.1 Å resolution (Table S1). CABA binds as observed previously. Oxovanadium appears bound in the active site, coordinated by the conserved 2-His-1-carboxylate facial triad (Fig. 3*C*). Strikingly, the oxygen atom of the mimic is not oriented toward CABA and is instead observed oriented away from the CABA binding site, in what would be an off-line configuration (Fig. 3*C*). This orientation is stabilized by a hydrogen bond made between the oxovanadium species and Arg310. This observation that the ScoE active site can accommodate an off-line metal-oxo species provides a mechanism for protection of a highly reactive Fe(IV)=O species as the active site undergoes the requisite conformational changes that must accompany a multistep reaction such as this one.

### Without a second Arg in the active site, α-KG binding appears to be weak

Next, we sought to structurally characterize the α-KG bound state. We obtained crystals by anaerobic cocrystallization of ScoE in the presence of α-KG and Fe(II), and the structure was solved to 1.85 Å resolution (Table S1). Weak electron density for a bidentate-bound molecule was observed at the metal center that was modeled as a single molecule of α-KG (Fig. S10). The molecule coordinates the metal center in a bidentate fashion and is also partially disordered, and electron density is not observed for C4 and C5 of this molecule, consistent with a lack of interactions being made between α-KG and protein side chains. This binding mode results in C4 and C5 of α-KG intruding upon the CABA binding site. Furthermore, a comparison to other α-KG-dependent dioxygenase active sites, such as that of TauD, reveals that ScoE lacks a conserved arginine (Arg266 in TauD) that is responsible for interacting with the 5-carboxylate moiety of α-KG (Fig. 3). Due to the unexpected nature of this α-KG binding mode, we have termed this configuration of the cosubstrate an off-site configuration.

After observing α-KG bound in an off-site configuration, we hypothesized that CABA binding may promote a conformational change to form a more stable α-KG binding site. To test this idea, we solved a crystal structure of ScoE in the presence of both α-KG and CABA to 1.45 Å resolution (Table S1). Unexpectedly, weak electron density was observed consistent with α-KG in the off-site and CABA in the previously described CABA binding site (Fig. S10). In order to prevent substantial steric clashes implied by the observed density, the CABA bound state and the off-site α-KG state were modeled as alternate conformations of each other, suggesting that the two states are mutually exclusive: CABA cannot bind to ScoE when α-KG is bound in this configuration and vice versa. Critically, we did not observe conformational changes in active site side chains that would indicate a new α-KG binding site (Fig. S10).

### A ScoE structure with tartrate bound reveals a new potential α-KG binding site

A vital insight into the binding of α-KG was gained from the recent reporting of another crystal structure of ScoE in the presence of CABA and tartrate, solved to 2.18 Å resolution by Chang and colleagues (13). As mentioned above, the CABA binding site is identical to the site we have observed in our 1.7 Å resolution structure. As a result of the precipitant solution used, a molecule of tartrate was observed bound to Fe(II), and unlike what we report above for α-KG, this tartrate molecule does not intrude upon the CABA binding site. Tartate binding in this ScoE structure is stabilized by a striking conformational change that repositions His299 and Arg157 such that their side chains are stacked with each other and are pointing toward the carboxylate moiety of tartrate (Fig. 3*D*). Modeling α-KG in place of tartrate demonstrates that the binding site defined by Arg157/His299 is large enough to both accommodate α-KG and preserve the interaction between this molecule and the side chain (Fig. S11). Additionally, this putative binding site for α-KG in ScoE is reminiscent of the α-KG binding site in TauD (Fig. 3, *B* and *D*, Fig. S11).

**Figure 6:**
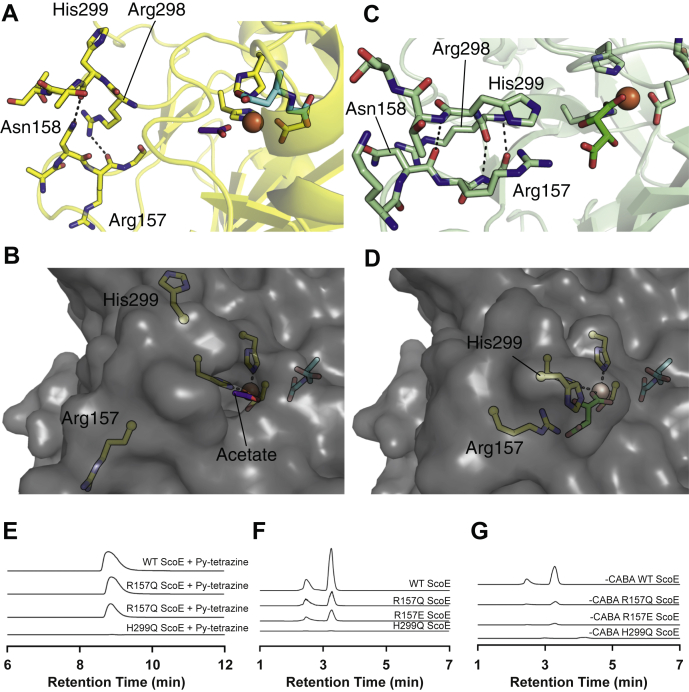
**Movement of Arg157 and His299 in the absence and presence of tartrate in the ScoE active site appears to be relevant for α-ketoglutarate binding.***A*, active site view of ScoE with CABA (*cyan*), Fe(II) (*orange sphere*), and acetate (*purple*) showing loop regions containing His299 and Arg 157, both flipped away from the active site. This loop conformation is stabilized by side chain to backbone hydrogen bonds (*black dashes*). *B*, surface of ScoE structure shown in panel *A*. When His299 and Arg157 are positioned away from the active site, the active site is open and accessible for αKG binding. *C*, active site view of ScoE (PDB: 6L6X) with Fe(II) (*orange sphere*) and tartrate (*green*), showing loop regions containing His299 and Arg157. His299 and Arg157 are positioned toward the active site and form a stacking interaction. This conformation is also stabilized by backbone interactions between the loops (*black dashes*). *D*, surface of ScoE structure shown in panel *C* (PDB: 6L6X). When His299 and Arg157 are flipped in, the active site is closed and no longer solvent accessible. *E*, extracted ion chromatograms corresponding to the production of Py-aminopyrazole (calculated:[M + H]+= 238.1087, observed:[M + H]+= 238.1095, 3.4 ppm error) from ScoE assays using wild-type ScoE and three single amino acid variants. In all cases, the calculated masses with a 10-ppm error tolerance were used. A reaction mixture comprised of 50 mM HEPES, pH 8, 500 μM CABA, 250 μM α-KG, 100 μM Apo-ScoE, and 90 μM (NH_4_)2Fe(SO_4_)_2_ was gently mixed and incubated for 10 min at room temperature, which was further quenched with 200 μl of 667 μM 3,6-di(pyridine-2-yl)-1,2,4,5-tetrazine dissolved in cold methanol. 3,6-Di-2-pyridyl-1,2,4,5-tetrazine reacts with ScoE product INBA to generate Py-aminopyrazole. *F*, extracted ion chromatograms corresponding to the production of succinate (calculated:[M + H]+= 117.0193, observed:[M + H]+= 117.0193, 0 ppm error) from ScoE assays using wild-type ScoE and three single amino acid variants. In all cases, the calculated masses with a 10-ppm error tolerance were used. The same reaction mixture as used in *E* was used for this analysis. *G*, extracted ion chromatograms corresponding to the production of succinate (calculated:[M + H]+= 117.0193, observed:[M + H]+= 117.0193, 0 ppm error) from ScoE assays lacking CABA using wild-type ScoE and three single amino acid variants. In all cases, the calculated masses with a 10-ppm error tolerance were used. A reaction mixture comprised of 50 mM HEPES, pH 8, 250 μM α-KG, 100 μM Apo-ScoE, and 90 μM (NH_4_)_2_Fe(SO_4_)_2_ was gently mixed and incubated for 10 min at room temperature, which was further quenched with 200 μl of 667 μM 3,6-di(pyridine-2-yl)-1,2,4,5-tetrazine dissolved in cold methanol.

In all crystal structures lacking tartrate both reported here and previously (4,13), Arg157 and His299 are both flipped away from the ScoE active site and do not interact with one another (Fig. 3*D*). These outward facing positions are observed even when CABA is present, indicating that this conformational change is not induced by CABA binding. The change itself is nontrivial with the repositioning of His299 and Arg157 side chains being a consequence of a dramatic alteration in the hydrogen bonding patterns between two strands in which side chains (Asn158, Arg298) to backbone hydrogen bonds are replaced by backbone-to-backbone hydrogen bonds (Fig. 6, *A*–*B*). With His299 and Arg157 pointed out, there is an access channel to the Fe(II), and with His299 and Arg157 pointing in, tartrate is sequestered in the active site (Fig. 6, *C*–*D*). We suspect that in solution, α-KG binding induces the conformational change that brings His299 and Arg157 into the active site, but that lattice contacts in our crystals between His299 and a neighboring molecule (Fig. S12) block the ability of α-KG to induce this conformational state, hindering our ability to visualize an Arg157/His299:α-KG interaction. We also suspect that the outward facing conformations of these residues are physiologically relevant, since there must be a route for α-KG to enter the active site and for succinate and CO_2_ to leave.

If we assume that Arg157 and His299 are the anchors for α-KG binding, then the question arises if both α-KG molecules that are required for INBA generation will interact with Arg157/His299. It is possible that prior to CABA binding, α-KG could bind to ScoE in the observed off-site binding mode (Fig. S9*A*) and react to form a protected, off-line Fe(IV)=O intermediate, similar to the off-line species that we observed in our ScoE structure with an oxovanadium (Fig. 3*C*). In this case, CABA binding would trigger a rearrangement of this intermediate and the first hydroxylation would occur (Fig. S13). The presence of a bound CABA-hydroxylated intermediate would preclude off-site α-KG binding due to the extensive steric clashes that this binding would entail, necessitating the movement of His299 and Arg157 into the active site to form a new α-KG binding site, allowing for the second α-KG-dependent reaction.

### Computational analyses suggest that movements of His299 and Arg157 are energetically feasible

To test the hypothesis that Arg157 and His299 could dynamically reposition and form interactions with the active site, we carried out classical molecular dynamics (MD) simulations. All structures were modeled to contain an off-line Fe(IV)=O intermediate and bound succinate, with His299 and Arg157 modeled in both inward positions from the structure of Chang and colleagues (13), only His299 or both in outward positions for the structure from the present work, as well as around nine favorable rotameric states supported by the local protein environment (Fig. S14 and Table S2). In total, over 3 μs of MD from these simulations suggests high flexibility of His299, which readily interconverts between the outward and inward orientations in almost all trajectories (Fig. S15). Although reorientation of Arg157 is less frequently observed in simulations, movement from the outward to the inward orientation in conjunction with His299 motion to an inward state does occur on the nanosecond timescale. Once in the inward orientations, both His299 and Arg157 are sufficiently proximal to form hydrogen bonds with the carboxylate tail of Fe-coordinated α-KG (Figs. S15 and S16).

### Substitution of residues Arg157 and His299 in the putative α-KG binding site leads to loss of enzyme activity

To probe the potential α-KG binding site described above, we generated three ScoE variants: H299Q, R157Q, and R157E, and investigated the ability of these enzyme variants to produce both INBA (Fig. 6*E*) and succinate (Fig. 6*F*) when reacted with α-KG and CABA. We found that H299Q completely eliminated production of INBA and succinate in these assays and R157 variants were less active compared with the wild-type counterpart (Fig. 6, *E* and *F*). These results suggest that H299 and R157 play a role in catalysis with H299 being the more important of the two residues. Given the positioning of these residues far from the CABA binding site, even when these residues are swung-in (Fig. 3*D*), we attribute the loss in activity of our ScoE variants to one or more of the reactions with α-KG. As discussed above, the first half-reaction with α-KG could occur using the off-site binding mode that exists prior to CABA binding (Fig. S13*B*). Thus, we next considered whether R157/H299 are required for the ability of ScoE to react with α-KG in the absence of CABA (Fig. 6*G*). Mimicking the results with CABA, we found that without CABA, H299Q ScoE was completely unable to produce succinate from α-KG and the Arg157 variants had decreased ability to produce succinate (Fig. 6*G*). These data suggest that there is only one binding site for α-KG and that this site is not the off-site binding mode observed when lattice contacts prevent His299/Arg157 movement, but rather, the binding site for α-KG is inducible and requires His299.

## Discussion

The discovery that isonitrile biosynthesis can occur by desaturation of a carbon–nitrogen bond (3) prompted immediate speculation as to the mechanism of the enzyme responsible. Bioinformatics, structural and biochemical characterizations firmly placed one of the responsible enzymes, ScoE, into the large and diverse Fe(II)/α-KG dioxygenase superfamily (3,4). Best known for catalyzing hydroxylation reactions, enzymes in this superfamily had not previously been associated with isonitrile formation, raising the question as to how ScoE is able to perform this unprecedented chemistry.

A key piece of missing data when we started this study was the stoichiometry of the ScoE reaction. In particular, we wanted to know the number of α-KG molecules that were required for isonitrile formation. These data were particularly important as we tried to understand structural data that showed electron density for α-KG overlapping with the CABA binding site (established both by our work and that of others) (4,13). We reasoned that α-KG could bind to an overlapping site in the absence of CABA, but if the reaction required two α-KG molecules, then there must be another α-KG binding site that was eluding detection. The alternative that a newly formed CABA intermediate must depart ScoE and later rebind to allow for a second α-KG reaction seemed unlikely. The experiments described here have now firmly established a stoichiometry of 2 α-KG molecules per INBA formed, leading us to believe that there must be an additional α-KG binding site on ScoE.

**Figure 7:**
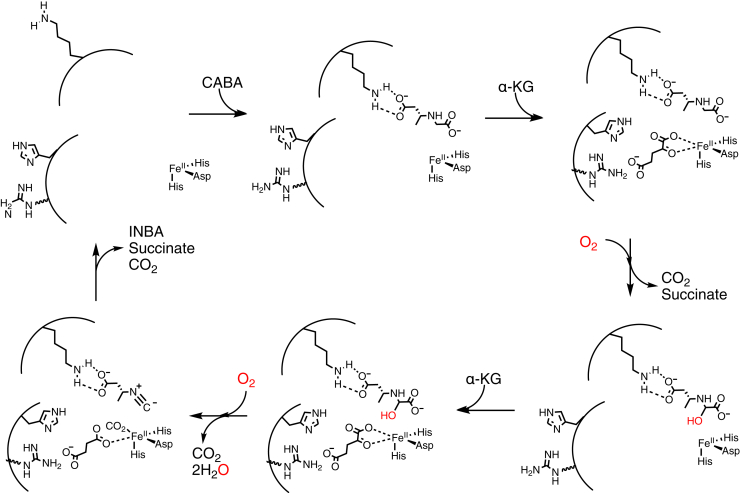
**Proposed inducible motions in ScoE that form binding sites for the cosubstrates during the ScoE reaction.** CABA binding is stabilized by a conformational change to bring the side chain of Lys193 into the active site to interact with one end of the CABA molecule. The side chains of His299 and Arg157 must swing a distance of 10 Å toward the active site to form the α-KG binding site and swing out again to release succinate and CO_2_. These side chains must swing back into the active site to form the binding site for the second molecule of α-KG. Upon formation of the INBA product, Lys193, His299, and Arg157 must all flip out of the active site to facilitate product release. Note: although CABA is shown as hydroxylated on C5, the site of the first (or second) hydroxylation is not firmly established.

Inspection of our ScoE structures revealed that the arginine typically associated with α-KG binding was missing. However, in a recent ScoE structure with tartrate (13), an arginine was found in the active site. This arginine (Arg157), along with His299, moved over 10 Å from a position on the surface of the protein into the active site. MD trajectories initialized from either crystal structure pose supported the expectation that interconversion between these two poses could occur at physiological temperatures. Conversion from the outward to inward poses of both His299 and Arg157 was directly observed on modest timescales (<250 ns) (Fig. S17). Here, we show that substitution of these residues abolished or decreased both the cleavage of α-KG into succinate and INBA production. It is surprising that His299, and not Arg157, is the more important of the two residues, given that an Arg residue is typically associated with α-KG binding and that Arg157 in the “in” position occupies the equivalent site as the α-KG-binding arginine in TauD (Fig. 3, *B* and *D*). Thus, it appears that ScoE differs from canonical α-KG-Fe(II) enzymes in that the residues involved in α-KG cleavage are mobile and are not exclusively arginine, but ScoE shares similarities, too. The discovery of this “inducible” α-KG binding site means that ScoE is similar to other family members (5) in that it appears to have an α-KG binding site that does not overlap with the substrate binding site, allowing ScoE to form a ternary complex (α-KG-CABA-ScoE). Formation of a ternary complex is a common feature in this superfamily (5) that limits the generation of a highly reactive Fe(IV)=O species before substrate is available for reaction. Enzymes that do not form ternary complexes rely on the formation of an off-line Fe(IV)=O species that is protected in the absence of substrate to prevent unwanted chemistry. Our structure with oxovanadium bound to ScoE indicates that the ScoE active site is capable of accommodating an off-line Fe(IV)=O species. However, we favor the mechanistic option that has the greater precedence in this superfamily, in which ScoE forms a ternary complex with both α-KG and CABA bound at the same time using the “inducible” site for α-KG binding (Fig. 7).

As far as we know, no other Fe(II)/α-KG dioxygenase has an “inducible” α-KG binding site with Arg157/His299 swinging 10 Å to create a binding site. The reason for this difference could lie in the nature of the two cosubstrates: CABA and α-KG. These molecules are very similar to each other, and an enzyme that binds both of them must have evolved so that CABA binds in its binding site and not where α-KG should bind and vice versa. Our structural data for ScoE in the presence of both α-KG and CABA, in which neither cosubstrate is bound with high occupancy, may reflect the design challenge represented by using such similar cosubstrates. Therefore, we speculate that to prevent CABA from binding between the Fe(II) and Arg157/His299, these residues are not permanently placed in the active site.

Another motivation for our structural studies was to determine which atoms on CABA might be accessible to the Fe(IV)=O cofactor for hydroxylation, given that the CABA modification sites have not been firmly established. The two atoms of CABA that are closest to the Fe ion are N and C5 (Fig 3*A*). From a structural prospective exclusively, C5 of CABA appears the most likely site of a first hydroxylation, which favors the mechanistic schemes shown in Figure 2, *i*–*ii*. However, the scheme shown in Figure 2*iii* cannot be ruled out as a small arrangement of CABA would make the N accessible. Additionally, this work has revealed that the residue that contacts N of CABA, Tyr96, is catalytically essential.

Although there is still much to learn about ScoE, these experiments have started to unravel ScoE’s enigmatic reaction mechanism. The reaction catalyzed by ScoE likely requires chemical steps that are unprecedented in the Fe(II)/α-KG dioxygenase enzyme superfamily and provides an orthogonal route for the biosynthesis of isonitriles compared to that of isonitrile synthases. Although the click chemistry that utilizes isonitriles is commonplace in biotechnology, there appears to be nothing commonplace about the chemistry involved in isonitrile biosynthesis.

## Experimental procedures

### Materials and methods

Plasmids were constructed for protein expression in *E. coli*. Individual genes were PCR amplified from genomic DNA and cloned into either pET24 B, pETCDFDuet-1, or pET30 by means of restriction enzyme digestion (ThermoFisher) and ligation with Quick T4 DNA Ligase (New England Biolabs). The primers used in this study are reported in Table M1. Plasmids were extracted using a Zyppy Miniprep Kit (Zymo Research) and confirmed by DNA sequencing at the UC Berkeley Sequencing Facility.

Point mutations of ScoE were generated by PCR using Phusion HighFidelity DNA Polymerase (Thermo Scientific) and the primers listed in Table M1. pET30- ScoE was used as a template for all mutants with the exception that R157Q was used as a template to generate R157E. Reactions were conducted according to the manufacturer’s protocol with supplied buffer using DMSO and each pair of primers. The PCR program began at 98 °C for 30 s, followed by 30 cycles of 72 °C for 10 s, 72 °C for 5 min, and final extension at 72 °C for 5 min. The template DNA was digested with 10 units of DpnI (Thermo Scientific) for 1 h at 37 °C, and the remaining PCR product was transformed into *E. coli* XL-1 Blue competent cells by heat shock. The introduction of point mutation was confirmed with DNA sequencing to yield pET30-ScoE-R157E, H299Q, Y101F, Y96F, Y97F, and R195Q.

### Overexpression and purification of ScoE for crystallization

The expression and purification for all proteins used in this study followed the same general procedure for His6-tag purification as detailed here. BL21 cells were grown at 37 °C in 1 l of LB in a shake flask supplemented with 50 μg/ml of kanamycin to an OD_600_ of 0.5 at 250 rpm. The shake flask was then placed over ice for 10 min and induced with 120 μM of isopropyl-β-D-thiogalactopyranoside (IPTG). The cells were then incubated for 16 h at 16 °C and 200 rpm to undergo protein expression. Subsequently, the cells were harvested by centrifugation (6371*g*, 15 min, 4 °C), and the supernatant was removed. The cell pellet was resuspended in 30 ml of lysis buffer (25 mM HEPES pH 8, 500 mM NaCl, 5 mM imidazole), and cells were lysed by sonication on ice. Cellular debris was removed by centrifugation (27,216*g*, 1 h, 4 °C), and the supernatant was filtered with a 0.45 μm filter before batch binding. Ni-NTA resin (Qiagen) was added to the filtrate at 1.5 ml/l of cell culture, and the samples were allowed to nutate for 1 h at 4 °C. The protein–resin mixture was loaded onto a gravity flow column. The flow-through was discarded and the column was then washed with approximately 25 ml of wash buffer (25 mM HEPES pH 8, 100 mM NaCl, 20 mM imidazole) and tagged protein was eluted in approximately 20 ml of elution buffer (25 mM HEPES pH 8, 100 mM NaCl, 250 mM imidazole). The whole process was monitored using a Bradford assay. Purified proteins were concentrated using Amicon Ultra spin filters to yield 3 ml of protein. ScoE was dialyzed at 4 °C using a 10 kDa Slide-A-Lyzer Dialysis Cassettes in 1 l of dialysis buffer (25 mM HEPES pH 8, 100 mM NaCl, 1 mM EDTA). The dialysis buffer was changed twice over 9 h and dialyzed overnight. After dialysis, ScoE was desalted to remove EDTA using a GE PD-10 column and eluted into a buffer containing 25 mM HEPES pH 8 and 100 mM NaCl. ScoE was concentrated using a 10 kDa Amicon Ultra spin filter until the protein concentration reached 40 mg/ml and 10% v/v glycerol was added. The proteins were flash frozen in liquid nitrogen and stored at −80 °C.

### Overexpression and purification of proteins for *in vitro* assays

The procedure for the overexpression and purification of proteins for *in vitro* biochemical assays followed the same general procedure as described in the previous paragraph, but with some minor differences mentioned here. BAP-1 chemical competent cells were utilized for overexpression and purification of ScoB. ScoA, B, and C proteins were concentrated in appropriately sized Amicon Ultra spin filters and exchanged in exchange buffer (25 mM HEPES pH 8, 100 mM NaCl). After three rounds of exchange, the purified proteins were flash frozen and stored as mentioned previously. Before utilization of ScoE for biochemical assays, a Bio-rad Bio-Gel P-6 gel column was equilibrated into 50 mM HEPES pH 8, 100 mM NaCl by following the manufacturer’s protocol and guidelines to remove EDTA from ScoE. The presence and purity of enzymes were assessed using SDS-PAGE (Fig. M1), and their concentration was determined using a NanoDrop UV-Vis spectrophotometer (ThermoFisher). The approximate protein yields were 10.4 mg/l for ScoA (158 kDa), 3.4 mg/l for ScoB (12 kDa), 17.2 mg/l for ScoC (57 kDa), 5.2 mg/l for ScoE (37 kDa), 4.5 mg/l for Y96F ScoE (37 kDa), 4.7 mg/l for Y97F ScoE (37 kDa), 5.0 mg/l for R195Q ScoE (37 kDa), 4.8 mg/l for Y101F ScoE (37 kDa), 4.6 mg/l for H299Q ScoE (37 kDa), 5.5 mg/l for R157Q ScoE (37 kDa), and 5.4 mg/l for R157E ScoE (37 kDa).

### ScoE reconstitution to yield holo-enzyme

Immediately before each assay, a Bio-Rad Bio-Gel P-6 gel column was equilibrated in exchange buffer containing 25 mM HEPES pH 8 and 100 mM NaCl. In total, 50 to 90 μl of ScoE was desalted to remove EDTA following the manufacturer’s protocol. ScoE was immediately added to the biochemical assay of interest and supplemented with ammonium iron(II) sulfate hexahydrate matching 90% of the protein concentration.

### Synthesis of isotope-labeled CABA

In general, the synthesis of CABA was followed as previously reported (4). Bromoacetic acid-2-^13^C was used for CABA-6-^13^C (5) synthesis (Fig. M2). The product was a colorless oily liquid and was confirmed by HRMS and ^1^H NMR (4). The product was a colorless oily liquid. Calculated for C_6_H_12_NO_4_ (6-^13^C) [M + H]^+^ 163.0794, found: 163.0794 (0 ppm error). ^1^H NMR (900 MHz, DMSO-_*d6*_) δ 3.77 (s, 2H), 3.51 (dd, J = 13.2, 6.6 Hz, 1H), 2.81 (dd, J = 16.6, 4.4 Hz, 1H), 2.48 (s, 1H), 1.23 (d, J = 6.6 Hz, 3H); ^13^C NMR (226 MHz, DMSO-_*d6*_) δ 171.70, 168.51, 50.16, 49.94, 45.23, 16.30.

### O_2_ consumption from ScoE *in vitro* reaction

To investigate O_2_ consumption from the ScoE *in vitro* reaction, a 0.5 ml single-turnover assay containing 50 mM HEPES pH 8, 500 μM CABA, 250 μM α-KG, 100 μM Apo-ScoE, and 90 μM (NH_4_)_2_Fe(SO_4_)_2_ was conducted in a sealed reaction chamber with an integrated oxygen electrode unit (Oxygraph Plus System, Hansatech Instruments, UK). The instrument was calibrated with 0.5 ml of 50 mM HEPES pH 8.0 at room temperature and a mixing rate of 50 RPM. The assay components were added to the reaction chamber except for the iron. The oxygen signal was allowed to equilibrate before addition of 90 μM (NH_4_)_2_Fe(SO_4_)_2_ with an airtight needle to initiate the reaction. Oxygen consumption was collected as a function of time, and the reaction was allowed to incubate until the profile reached a steady state. The difference between the starting and final oxygen concentrations was used to estimate O_2_ consumption. Two 100 μl aliquots of the reaction mixture were quenched with 200 μl of cold methanol and 200 μl of 667 μM 3,6-di(pyridine-2-yl)-1,2,4,5-tetrazine dissolved in cold methanol, respectively for coupled stoichiometry quantification experiments.

### Detection and quantification of isonitrile butanoic acid (INBA) using tetrazine click reaction

A ScoE *in vitro* assay containing the same reaction components as mentioned previously was quenched with 200 μl of 667 μM 3,6-di(pyridine-2-yl)-1,2,4,5-tetrazine dissolved in cold methanol, gently mixed, and incubated for 1 h at room temperature. The reaction mixture was vortexed briefly and centrifuged to remove aggregated protein. In total, 100 μl standards containing 50 mM HEPES pH 8, 500 μM CABA, 250 μM α-KG, and varying concentrations of 3,5-di(pyridine-2-yl)-1H-pyrazol-4amine (Py-aminopyrazole) were quenched using the same quench method to develop a standard curve using LC-HRMS. LC-HRMS analysis was performed on an Agilent Technologies 6545 Accurate-Mass Q-TOF LC-MS instrument and an Eclipse Plus C18 column (100 x 4.6 mm). Chromatography was performed using a linear gradient of 10 to 50% acetonitrile (vol/vol) with 0.1% formic acid over 12 min in water with 0.1% formic acid (vol/vol) at a flow rate of 0.5 ml/min. A Py-aminopyrazole standard was synthesized and characterized by NMR as previously described (14) and was observed by LC-HRMS as [M + H]^+^=238.1095 (calculated [M + H]^+^: 238.1087, 3.4 ppm error). A standard curve was used to quantify the production of INBA.

### Quantification of succinate production, CABA consumption, and α-KG consumption

A ScoE *in vitro* assay containing the same reaction components as mentioned previously was quenched with cold methanol, gently mixed, vortexed, and centrifuged to remove the aggregated protein. In total, 100 μl standards containing 50 mM HEPES pH 8 and varying concentrations of CABA, α-KG, and succinate were quenched with 200 μl of cold methanol to develop a standard curve using LC-HRMS. LC-HRMS analysis was performed on an Agilent Technologies 6545 Accurate-Mass Q-TOF LC-MS instrument and an Eclipse Plus C18 column (100 × 4.6 mm). Chromatography was performed using a linear gradient of 10 to 50% acetonitrile (vol/vol) with 0.1% formic acid over 12 min in water with 0.1% formic acid (vol/vol) at a flow rate of 0.5 ml/min. Extracted ion chromatograms (EICs) for CABA, α-KG, and succinate were generated using the following m/z: [M + H]^+^=162.0761 for CABA, [M − H]^−^=145.0142 for α-KG, and [M − H]^−^=117.0193 for succinate. The following m/z values were observed: [M + H]^+^=162.0763 for CABA (1.2 ppm error), [M − H]^−^=145.0142 for α-KG (0 ppm error), and [M − H]^−^=117.0193 for succinate (0 ppm error). Standard curves for CABA, α-KG, and succinate were utilized to quantify their respective consumption and production

### H_2_O_2_ detection methods

To detect H_2_O_2_, the 0.5 ml ScoE enzymatic assay mentioned earlier was conducted with the same oxygen electrode unit. After the concentration of oxygen reached equilibrium, 120 U/ml of catalase was added to the reaction chamber. No appreciable increase of oxygen was observed when compared with adding solely water or buffer, indicating that no H_2_O_2_ was present in the reaction mixture. In addition, H_2_O_2_ was again undetected using the Amplex Red Hydrogen Peroxide/Peroxidase Assay Kit by following the manufacturer’s instructions.

### Formate detection methods

A 200 μl *in vitro* assay containing 50 mM HEPES pH 8, 500 μM CABA, 250 μM α-KG, 100 μM Apo-ScoE, and 90 μM (NH_4_)_2_Fe(SO_4_)_2_ was performed and incubated at room temperature for 10 min. This assay was adapted from previous work (2). The reaction was quenched with 400 μl of cold MeOH and 100 μl of the supernatant was reacted with 10 μl of 290 mM 1-ethyl-3-(3-dimethylaminopropyl)carbodiimide (EDC) and 10 μl of 120 mM 2-nitrophenylhydrazine (dissolved in 250 mM HCl). The reaction was incubated at 60 °C for 15 min and centrifuged. The supernatant was analyzed with LC-HRMS analysis on an Agilent Technologies 6545 Accurate-Mass Q-TOF LC-MS instrument and an Eclipse Plus C18 column (100 × 4.6 mm). Chromatography was performed using a linear gradient of 10 to 50% acetonitrile (vol/vol) with 0.1% formic acid over 12 min in water with 0.1% formic acid (vol/vol) at a flow rate of 0.5 ml/min. A standard curve of all the previously mentioned components (except enzyme) was constructed using different sodium formate concentrations. Extracted ion chromatograms were constructed with [M + H]^+^= 182.0561 (observed: [M + H]^+^= 182.0558, 1.6 ppm error). Additionally, a Sigma-Aldrich Formate Assay Kit was utilized according to the manufacturer’s protocol, resulting in no detectable amounts of formate.

### CO detection method

A 200 μl *in vitro* assay containing 50 mM HEPES pH 8, 500 μM CABA, 250 μM α-KG, 100 μM Apo-ScoE, and 90 μM (NH_4_)_2_Fe(SO_4_)_2_ was performed and incubated at room temperature for 10 min in a sealed septum. This assay was adapted from previous work (18). A solution of myoglobin and sodium dithionite was added to give 10 μM and 20 mM final concentrations, respectively, and the reaction mixture was allowed to incubate for 10 min. The septum was immediately removed, and the absorption spectrum was recorded right after (150 μl) using a flat-bottom 96-well plate and Biotek plate reader. The Soret band remained at 434 nm for both the reaction-Fe control and -ScoE control, supporting the absence of CO.

### GC-MS experiments

For the detection of CO_2_ and CO, a 4.5 ml of reaction mixture contained 50 mM HEPES pH 8, 90 μM (NH_4_)_2_Fe(SO_4_)_2_, 2 mM (8 or unlabeled), 1 mM α-KG (1,2,3,4- ^13^C labeled or unlabeled), and 100 μM ScoE. The reaction was performed in a 10-ml sealed headspace vial (Agilent) and initiated by adding the iron. One milliliter of the headspace gas was acquired using a gastight syringe (Hamilton) and injected into Agilent 5977A GCMS system equipped with a HP-5 ms column. Injector temperature was set at 120 °C, and helium was used as the carrier gas at a flow rate of 3 ml/min. The temperature gradient was as follows: 40 to 100 °C over 5 min. The mass spectrometer was operated in electron ionization mode with automatically tuned parameters, and the acquired mass range was m/z = 15 to 100. The CO_2_ and CO signals were confirmed using authentic standards, and the production of ^13^CO_2_ and ^13^CO was determined by extracting m/z = 45 and 29, respectively.

### Crystallization

Crystals, which were used in the soaking experiments described below as well as to obtain the structure of Fe(II)-ScoE with an off-site α-KG, were grown by the sitting drop vapor diffusion technique at 24 °C in an anaerobic environment (95% Ar, 5% H_2_; Coy Laboratory Products, Inc), following optimization of the previously published precipitant solution (4). A 1 μl aliquot of protein solution (8 mg/ml metal-chelated ScoE, 50 mM HEPES pH 8, 100 mM NaCl, and 1 mM α-KG) was added to 2 μl of precipitant solution (205 mM sodium acetate, 100 mM Tris pH 8.5, 24% (w/v) PEG 4000) supplemented with 250 μM Fe(II)Cl_2_ in a 24-well sitting drop tray. Crystals formed within 1 week and reached a maximum size of approximately 50 μm after approximately 2 weeks. Paraffin oil was used for cryoprotection during crystal harvesting. Crystals were manually looped and streaked through paraffin oil then flash-frozen in liquid N_2_.

To obtain the structure of (*R*)-3-((carboxymethyl)amino)butanoic acid (CABA)-Fe(II)-ScoE, crystals that were grown as described above were transferred into 2 μl of a soaking solution containing 205 mM sodium acetate, 100 mM Tris pH 8.5, 24% (w/v) PEG 4,000, 1 mM CABA, and 250 μM Fe(II)Cl_2_ and lacking α-KG at 24 °C in an anaerobic environment (95% Ar, 5% H_2_; Coy Laboratory Products, Inc).

To obtain a structure of CABA-Fe(II)-ScoE with α-KG, crystals that were grown as described above were transferred into 2 μl of soaking solution containing 205 mM sodium acetate, 100 mM Tris pH 8.5, 24% (w/v) PEG 4,000, 1 mM CABA, 1 mM α-KG, and 250 μM Fe(II)Cl_2_ [room temperature] in an anaerobic environment. Crystals were soaked for 24 h prior to cryoprotection by manual looping and streaking through paraffin oil followed by flash-freezing in liquid N_2_. This soaking procedure leads to the structure of CABA-Fe(II)-ScoE with off-site α-KG.

To obtain the structure of CABA-oxovanadium-ScoE, ScoE was cocrystallized with CABA and oxovanadium using the sitting drop vapor diffusion technique at 24 °C in aerobic conditions. A 1 μl aliquot of protein solution (8 mg/ml metal-chelated ScoE, 50 mM HEPES pH 8, 100 mM NaCl, 1 mM succinate, 1 mM CABA, and 1 mM vanadium (IV) sulfate) was added to 2 μl of precipitant solution (205 mM sodium acetate, 100 mM Tris pH 8.5, 24% (w/v) PEG 4000). Crystals formed within 1 week and reached a maximum size after approximately 2 weeks. Paraffin oil was used as a cryoprotectant for crystal harvesting. Crystals were manually looped and streaked through paraffin oil then flash-frozen in liquid N_2_.

### Data collection and processing

Data for structures of CABA-Fe(II)-ScoE; and CABA-Fe(II)-ScoE with off-site α-KG; Fe(II)-ScoE with off-site α-KG were collected at the Advanced Photon Source (Argonne, Illinois, USA) on beam line 24ID-C using a Pilatus 6M pixel array detector at a temperature of 100 K at a wavelength of 0.9791 Å in a single 180° wedge and were processed, indexed, integrated, and scaled in XDS (23). Data for the structure of CABA-oxovanadium-ScoE were collected at the Advanced Photon Source (Argonne, Illinois, USA) on beam line 24ID-E using an Eiger 16M pixel array detector at a temperature of 100 K at a wavelength of 0.9791 Å in a single 360° wedge and indexed, integrated, and scaled in XDS (23). Resolution cutoffs were determined based on a combination of R_merge_, CC_1/2_, I/σ, and data completeness in the highest resolution bin.

### Structure determination and refinement

All ScoE structures were solved by molecular replacement using Phaser (24) to the full extent of resolution using the previously published 1.8 Å resolution structure of ScoE (PDB 6DCH) (4) with ligands and metals removed. Molecular replacement resulted in a solution with one ScoE protomer within the asymmetric unit for all data sets. Test sets for R_free_ calculations were chosen to be comprised of the same reflections as were used for refinement of the previously published crystal structure of ScoE for all data sets. Initial rounds of refinement included simulated annealing to reduce model bias from molecular replacement. Iterative rounds of model building were performed in Coot (25), followed by positional refinement and individual *B*-factor refinement performed in Phenix (26). Mononuclear Fe(II) and oxovanadium were modeled in early stages of refinement based on local environment, coordination geometry, and positive $F_{O}-F_{C}$ difference density. Restraint files for CABA and α-KG were generated using the Grade web server (27). Positive $F_{O}-F_{C}$ difference density was used to assist modeling of CABA, α-KG, chloride, and oxovanadium. Density for α-KG was not of good quality in any structure. When density was present that could not be explained by the presence of water molecules or other compounds in the crystallization buffer, α-KG was modeled and refined. In no case did the refinement indicate full occupancy for α-KG, as strong negative $F_{O}-F_{C}$ difference density would appear when α-KG was refined at full occupancy. Thus α-KG was refined at partial occupancy in one conformation with extra positive $F_{O}-F_{C}$ difference density modeled as water molecules at partial occupancy, although this positive difference density might also indicate alternative α-KG positions. Regardless, we believe that lattice contacts prevent α-KG from assuming its relevant position in our structures and that each α-KG is bound in an off-site position (see main text), which would explain the poor density. The final occupancies of these molecules were calculated by fixing the B-factors to those of atoms in the surrounding environment followed by refinement of partial occupancies. Remaining water molecules were added manually during advanced stages of refinement. Final models for all structures were confirmed with simulated annealing composite omit maps generated in Phenix. All structures contain residues 28 to 323 (of 326 residues). All software used for refinement was compiled by SBGrid. Final refinement statistics can be found in Table S1. All figures were made using the PyMOL molecular graphics system version 2.0.7 (Schrodinger, LLC).

### Computational analysis

#### Protein structure and preparation

The present work’s crystal structure of ScoE with Fe, α-KG, and CABA was modified to model the Fe(IV)-oxo intermediate with succinate bound using Avogadro (28) and PyMOL (Schrodinger) (structure labeled CS1). This structure represents the outward orientation of Arg157 and His299, in which these residues are pointing away from succinate’s tail. The charge state of ScoE was assigned using the H++ web server (29, 30, 31) assuming a pH of 7.0 with all other defaults applied. After manual charge assignment of residues adjacent to cofactors/substrates, ScoE has a net charge of −15. Two further structures (CS2 and CS3) were prepared by aligning CS1 to another crystal structure of ScoE (PDB: 6L6X (13)) in PyMOL, the latter representing Arg157 and His299 in inward state, pointing toward the succinate tail. For CS2, the coordinates of Arg157 from the crystal structure (PDB: 6L6X) were aligned to CS1. For CS3, the coordinates of both Arg157 and His299 from the latter structure (PDB: 6L6X) were copied onto CS1. Consequently, CS1 represents both Arg157 and His299 in outward state, CS2 represents Arg157 in inward and His299 in outward state, and CS3 represents both Arg157 and His299 in inward state (Fig. S13).

To obtain further different configurations of Arg157, PyMOL’s mutagenesis tool was employed to generate different rotamers of Arg157 residue, starting with CS1. PyMOL found 22 backbone-dependent and 81 backbone-independent rotamers for Arg157 (and none for His299). Based on visual inspection and closest distance between the tail of succinate and side chain of Arg157, three backbone-dependent (BD6, BD20, BD21) and six backbone-independent (ID16, ID21, ID29, ID38, ID39, and ID52) rotamers were selected for collecting dynamics (Table S2).

For nonstandard residues (succinate and CABA), we use the generalized AMBER force field (GAFF) (32) with restrained electrostatic potential (RESP) (33) charges obtained at the Hartree-Fock/6-31G∗ (34) level using Gaussian/16 (35). AMBER’s Python utility for the Metal Center Parameter Builder (MCPB.py) (36) was used to obtain force field parameters to describe the metal active site. The QM geometry optimization, force constant calculation, and RESP charge calculation needed for MCPB.py were performed using Gaussian/16 (35) with functional UB3LYP (37, 38, 39) and basis set LANL2DZ effective core potential (40) on Fe and 6-31G∗ (34) for the remaining atoms.

The protein structures were solvated in a periodic rectangular prism box with at least a 10 Å buffer of TIP3P (41) water and neutralized with 15 Na^+^ counterions for a total simulation of 42,923 atoms (4741 protein/substrate atoms).

#### MM equilibration and dynamics

The structures were equilibrated with MM MD using the GPU-accelerated PMEMD code in AMBER/18 (42). Equilibration steps were: i) restrained (1000 steps) and unrestrained (2000 steps) minimizations, ii) 10-ps NVT heating to 300 K with a Langevin thermostat with collision frequency of 5.0 ps^−1^ and a random seed, and iii) 1-ns NpT equilibration using the Berendsen barostat with a pressure relaxation time of 2 ps. Production dynamics were collected for at least 250 ns for each structure (Table S2). The SHAKE algorithm (43) was applied with a 2-fs timestep for all MD, and the particle mesh Ewald method was used for long-range electrostatics with a 10-Å electrostatic cutoff.

#### Analysis of MD trajectories

Snapshots spaced 1 ns apart were analyzed for all configurations using the cpptraj utility in AMBER/18 (42). For each trajectory, four distances were computed between the two oxygen atoms of succinate’s COO^−^ tail and the two nitrogen atoms on side chains of Arg157 (the terminal NH_2_ groups) and His299 (the nitrogen atoms on sidechain ring). The minimum of the former four distances was labeled d_R157_ and the minimum of the latter four distances was labeled d_H299_ (Figs. S14 and S15).

## Data availability

Atomic coordinates and structure factors have been deposited in the Protein Data Bank (www.rcsb.org) under the following accession codes: 6XN6 (CABA-Fe(II)-ScoE), 6XO3 (Fe(II)-ScoE with off-site α-KG), 6XOJ (CABA-Fe(II)-ScoE with off-site α-KG), and 6XPA (CABA-oxovanadium-ScoE).

## Conflicts of interest

The authors declare that they have no conflicts of interest with the contents of this article.
