# Supplementary material for: Biochemical and crystallographic investigations into isonitrile formation by a nonheme iron-dependent oxidase/decarboxylase
Source: J Biol Chem. 2021 Jan 7;296:100231. doi: 10.1074/jbc.RA120.015932 (PMC7949033; doi:10.1074/jbc.RA120.015932)
Supplement: Supplemental Figures and Tables [file mmc1.pdf]

Biochemical and Crystallographic Investigations into Isonitrile Formation by a Non-Heme Iron-Dependent Oxidase/Decarboxylase

SI Figures and Tables

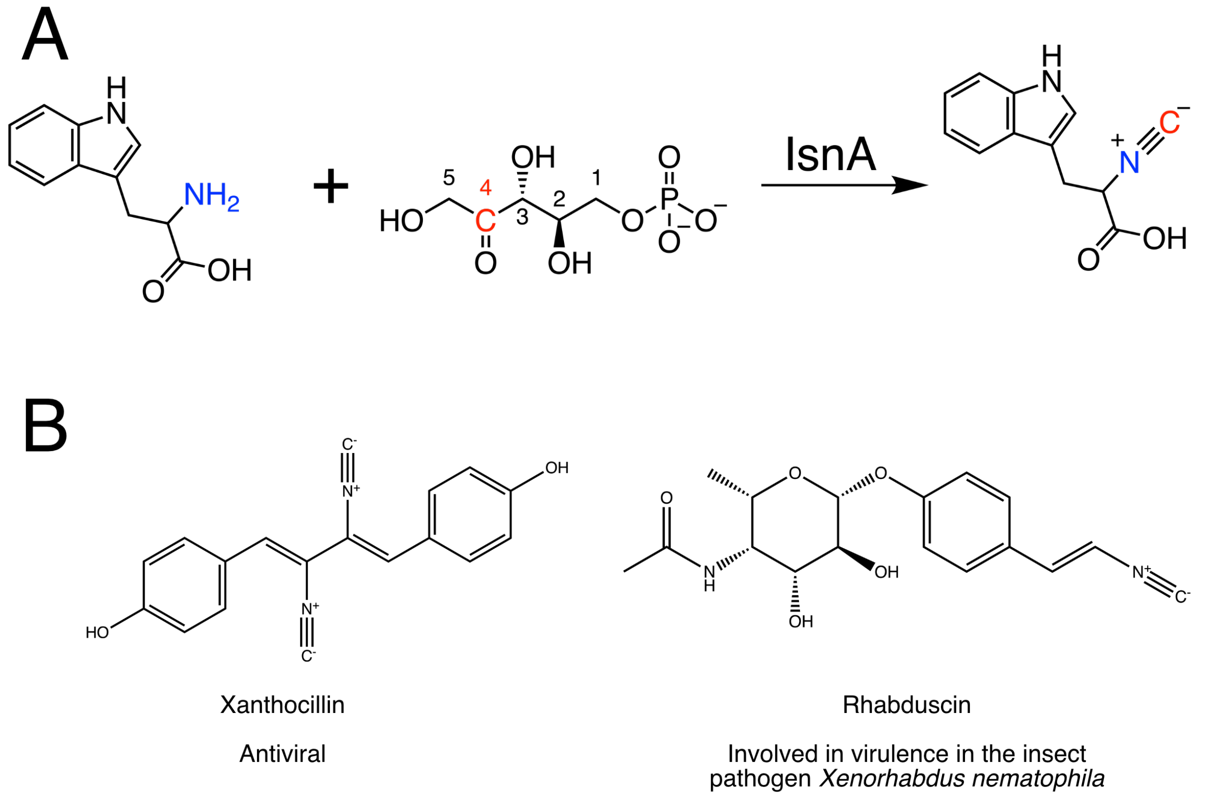

Figure S1: The IsnA biosynthesis of isonitriles and some isonitrile natural products. A) The IsnA enzymes generate isonitriles using the nitrogen of the α-amino group on tryptophan (shown in blue) or tyrosine, and a carbon atom from ribulose-5-phosphate (atom shown in red). **B)** Two natural products containing isonitriles.

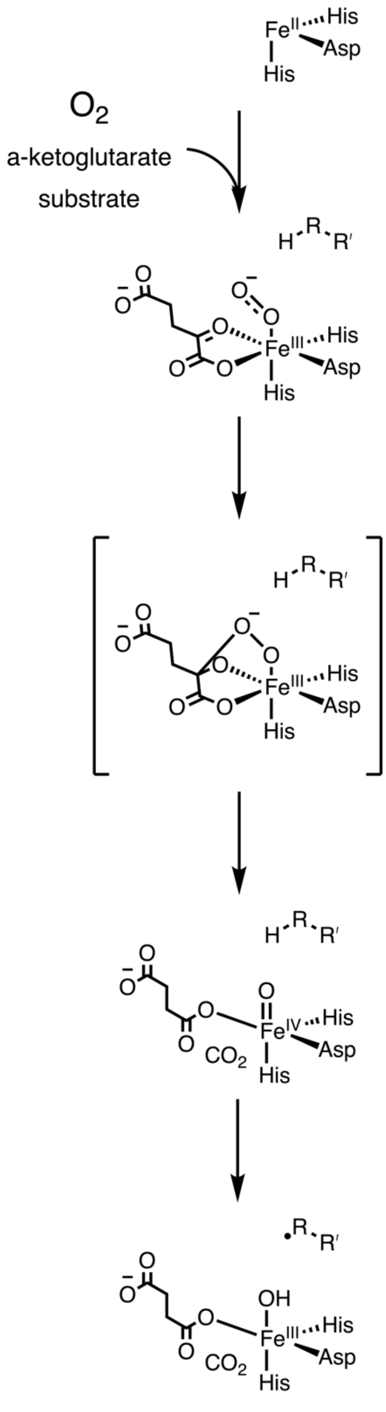

Figure S2: Mechanism of substrate radical formation in Fe(II)/αKG-dioxygenases. All members of the enzyme family are believed to share these steps. Prior to binding of α-KG and molecular oxygen, the three open coordination sites of Fe(II) are occupied by water molecules. Oxygen is thought to perform nucleophilic attack on α-KG to generate succinate, CO_2, and the Fe(IV)-oxo intermediate, but this step (shown in brackets) has not been directly observed.

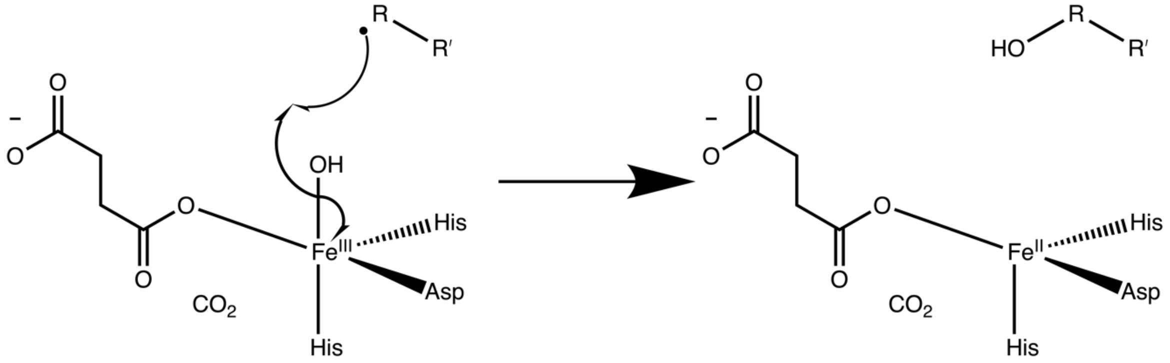

Figure S3: Proposed mechanism of hydroxyl rebound to complete the hydroxylation mechanism.

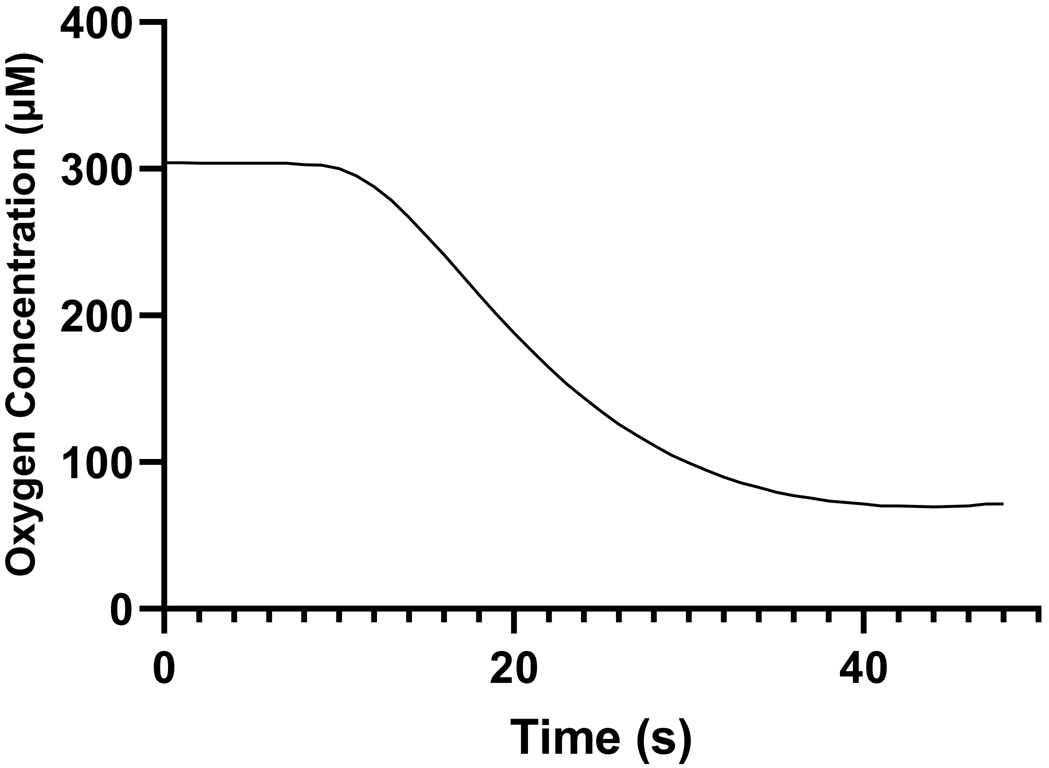

Figure S4: Representative plot of a measurement of oxygen consumption over the course of the ScoE *in vitro* reaction. The oxygen level was recorded using an Oxygraph Hansatech Probe. A reaction mixture comprised of 50 mM HEPES, pH 8. 500 µM CABA, 250 µM α-KG, and 100 µM Apo-ScoE were added to the reaction vessel. 90 µM $(NH_4)_2Fe(SO_4)_2$ were added to initiate the reaction, causing a drop in oxygen concentration as the ScoE reaction consumes oxygen. The concentration difference between both plateaus corresponds to the oxygen consumption from the ScoE reaction.

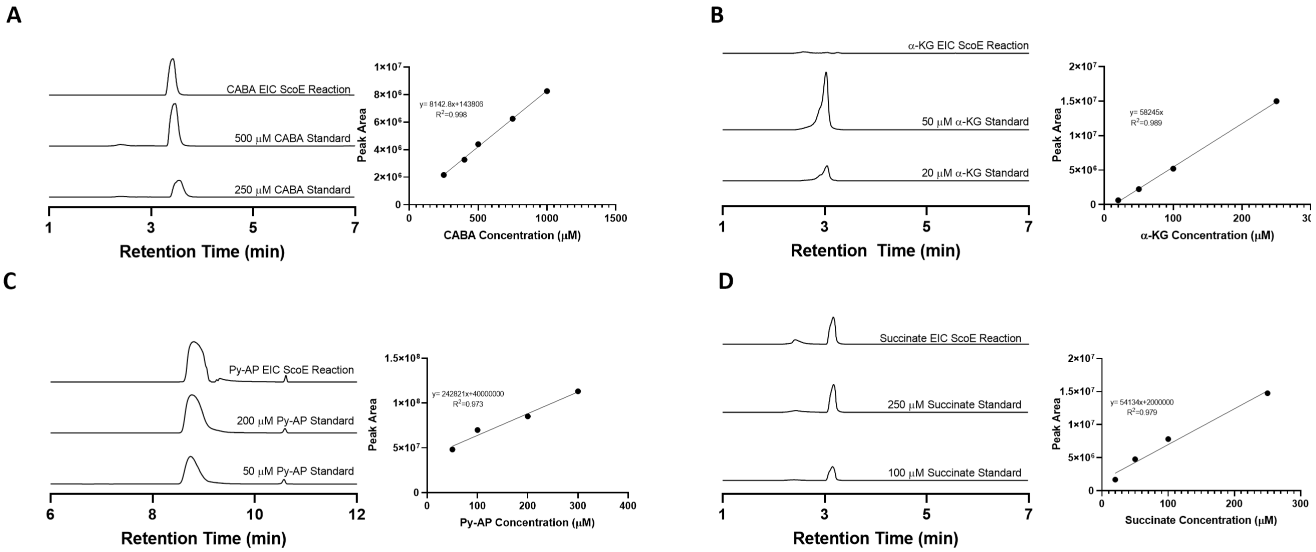

Figure S5: Quantification of CABA, α-KG, succinate, and INBA from ScoE *in vitro* reaction. Two 100 µL aliquots of the reaction mixture from the Oxygraph Hansatech probe experiments were analyzed for stoichiometry experiments. The first aliquot was quenched with 200 µL of cold methanol for quantification of CABA, α-KG, and succinate. The second aliquot was quenched with 200 µL of 667 µM 3,6-di(pyridine-2-yl)-1,2,4,5-tetrazine dissolved in cold methanol to quantify INBA production by detection of Py-aminopyrazole. Standards were composed of 100 µL samples containing varying concentrations CABA, α-KG, succinate, and Py-aminopyrazole, and quenched with the previous two methods. All samples were analyzed with LC-HRMS. **A)** Extracted ion chromatograms of CABA for one representative reaction and two standards (calculated: $[M+H]^+$= 162.0761, observed: $[M+H]^+$= 162.0761, 1.2 ppm error). **B)** Extracted ion chromatograms of α-KG for one representative reaction and two standards (calculated: $[M-H]^-$= 145.0142, observed: $[M-H]^-$= 145.0142, 0 ppm error). **C)** Extracted ion chromatograms of Py-aminopyrazole for one representative reaction and two standards (calculated: $[M+H]^+$= 238.1087, observed: $[M+H]^+$= 238.1095, 3.4 ppm error). **D)** Extracted ion chromatograms of succinate for one representative reaction and two standards (calculated: $[M-H]^-$= 117.0193, observed: $[M-H]^-$= 117.0193, 0 ppm error). In all cases, the standard curve used for quantification is shown and the calculated masses with a 10-ppm error tolerance were used.

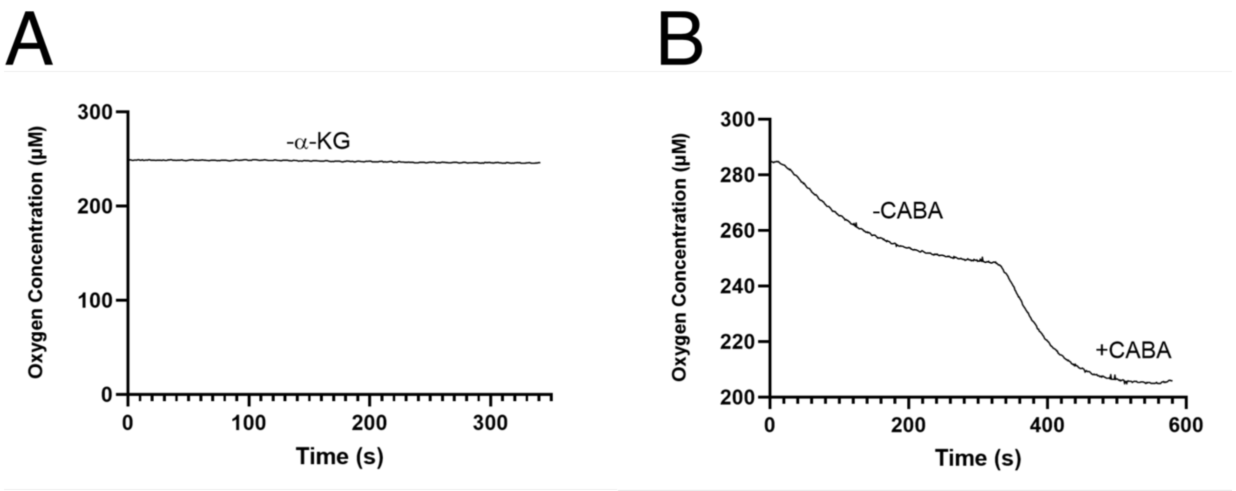

Figure S6: Oxygen consumption measurements in the presence and absence of α-KG and CABA. A) Oxygen consumption measured in the absence of α-KG. The reaction mixture was comprised of 50 mM HEPES, pH 8. 500 µM CABA and 100 µM Apo-ScoE were added to the reaction vessel. 90 µM $(NH_4)_2Fe(SO_4)_2$ was added to initiate the reaction. **B)** Oxygen consumption measured with respect to the presence of CABA. A reaction mixture comprised of 50 mM HEPES, pH 8. 250 µM α-KG and 100 µM Apo-ScoE were added to the reaction vessel and, once the oxygen signal plateaued, 90 µM $(NH_4)_2Fe(SO_4)_2$ were added to initiate the reaction. CABA was added at approximately 360 seconds.

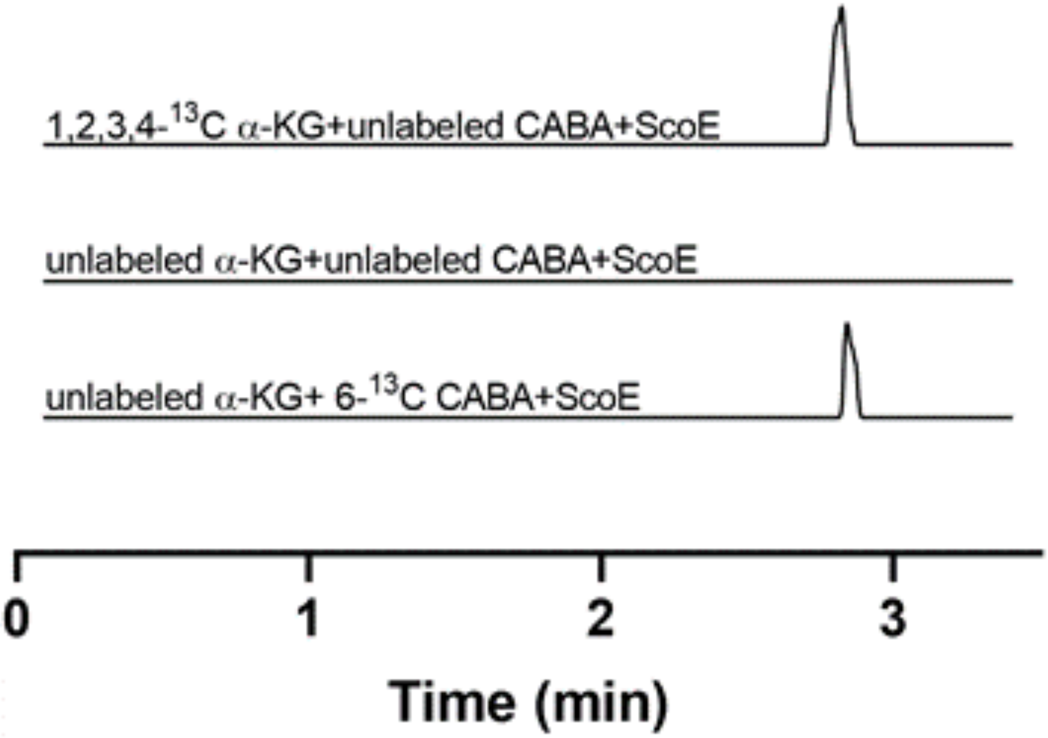

Figure S7: GC-MS detection of [^{13}C]-labeled carbon dioxide generated by ScoE using either [^{13}C]- labeled α-KG or [^{13}C]-CABA. Extracted ion chromatograms demonstrating the production of [^{13}C]-CO_2 (calculated:m/z= 45, observed:m/z= 45, 0 ppm error). Top: [^{13}C]-CO_2 generated from a ScoE assay using [1,2,3,4-^{13}C]-labeled α-KG. Middle: Control ScoE reaction with no ^{13}C label. Bottom: [^{13}C]-CO_2 generated from a ScoE assay using [6-^{13}C]-labeled CABA.

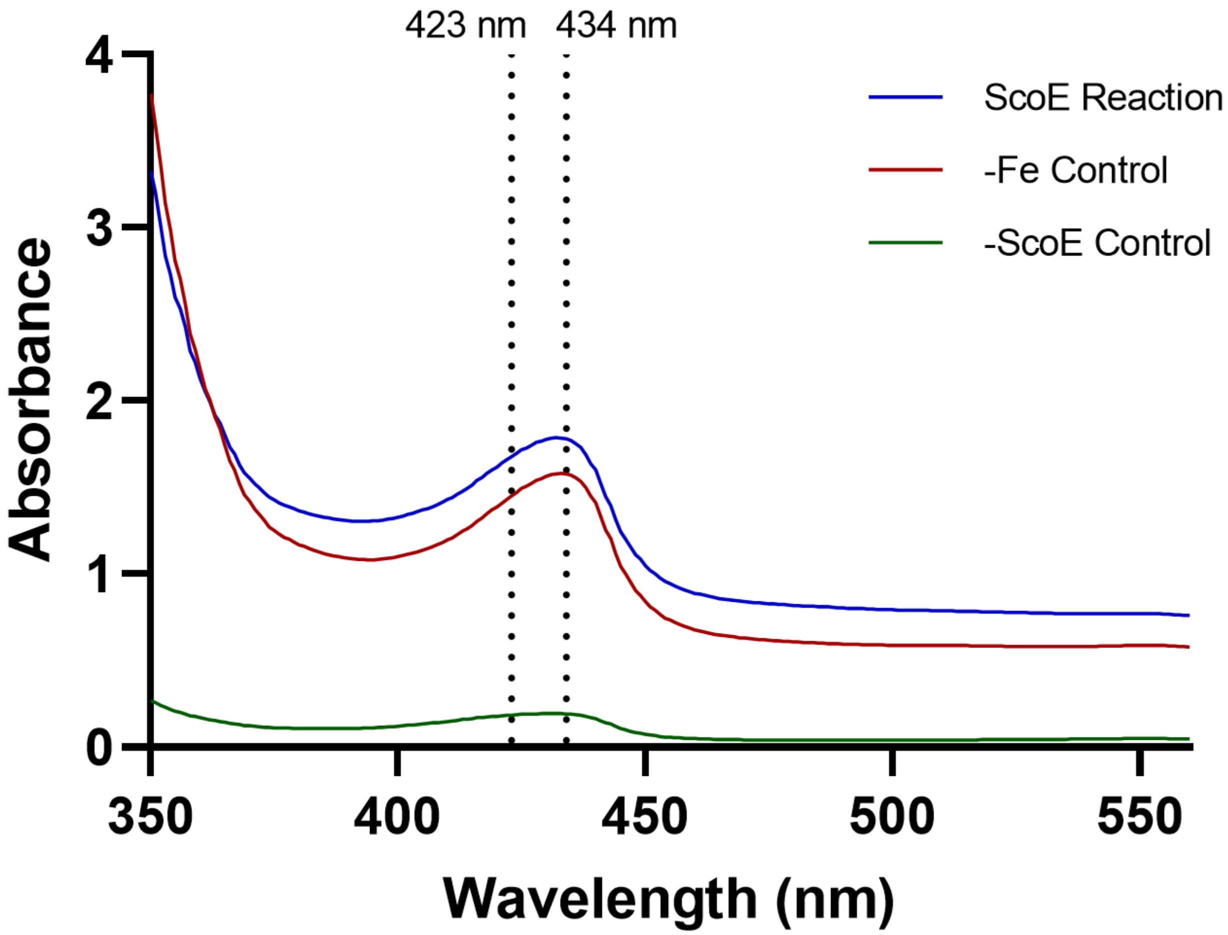

Figure S8: CO detection assay with sodium dithionite-reduced myoglobin. The presence of CO was assessed by its ability to bind to the Fe(II) site of oxygen-deprived myoglobin. A 200 µL biochemical assay containing 50 mM HEPES, pH 8, 500 µM CABA, 250 µM α-KG, 100 µM Apo-ScoE, and 90 µM $(NH_4)_2Fe(SO_4)_2$ was incubated at room temperature for 10 minutes in a sealed septum. A solution of 10 µM myoglobin and 20 µM sodium dithionite was subsequently added, and the reaction mixture was allowed to incubate for 10 minutes at room temperature before obtaining the absorption spectra. A shift in the Soret band from 434 nm to 423 nm suggests the presence of CO, which is clearly not shown here. Negative controls lacking ScoE and $(NH_4)_2Fe(SO_4)_2$ are shown as well.

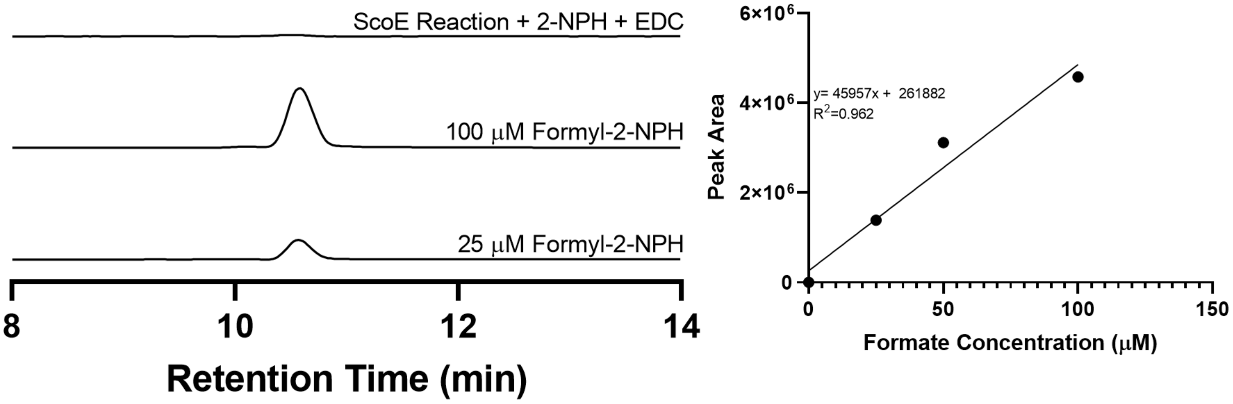

Figure S9: Formate detection via formate-adduct assay and LC-HRMS. 2-nitrophenyl hydrazine (2-NPH) can react with formate to produce formyl-2-NPH with the aid of 1-ethyl-3-(3-dimethylaminopropyl)carbodiimide (EDC) to produce an easily detectable formate adduct product. A 200-µL biochemical assay containing 50 mM HEPES, pH 8, 500 µM CABA, 250 µM α-KG, 100 µM Apo-ScoE, and 90 µM $(NH_4)_2Fe(SO_4)_2$ was incubated at room temperature for 10 minutes. The reaction was quenched with 400 µL of cold MeOH and 100 µL of the supernatant were reacted with 10 µL of 290 mM EDC and 10 µL of 120 mM 2-NPH (dissolved in 250 mM HCl). The reaction was incubated at 60 ˚C for 15 minutes and the resulting reaction mixture was analyzed with LC-HRMS analysis to directly detect formyl-2-NPH. Formate standards containing 50 mM HEPES, pH 8.0, 500 µM CABA, and 250 µM α-KG were prepared for quantification. The extracted ion chromatograms shown correspond to formyl-2-NPH (calculated: $[M+H]^+$= 182.0561, observed: $[M+H]^+$= 182.0558, 1.6 ppm error) for a representative ScoE reaction and two formate standards, demonstrating no formate detected from the ScoE reaction. In all cases, the standard curve used for quantification is shown and the calculated masses with a 10-ppm error tolerance were used.

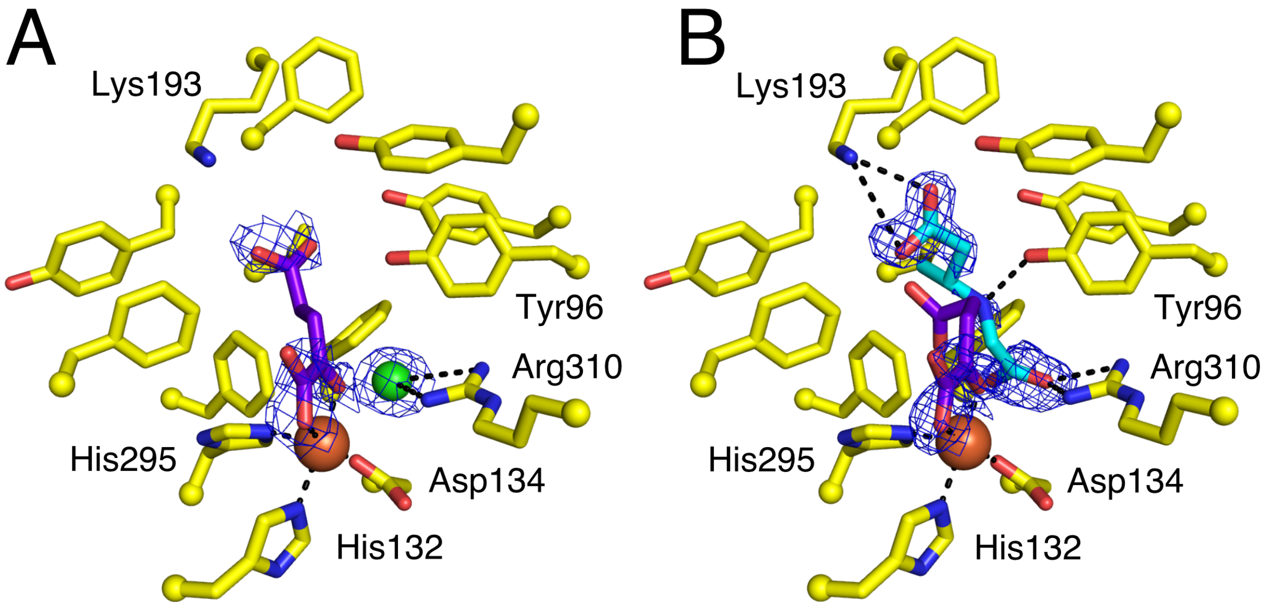

Figure S10: ScoE active sites with α-KG observed in the off-site orientation. A) ScoE with α-KG (purple) bound to Fe(II) (orange sphere) in the off-site configuration, and a chloride ion (green sphere) that interacts with Arg310 when CABA is not present. **B)** ScoE with CABA (cyan) bound and α-KG (green) bound to Fe(II) (orange sphere) in the off-site orientation that intrudes on the CABA binding site. In order to prevent clashes, the two co-substrates were modeled as alternate conformations. Thr127 is not shown for clarity.

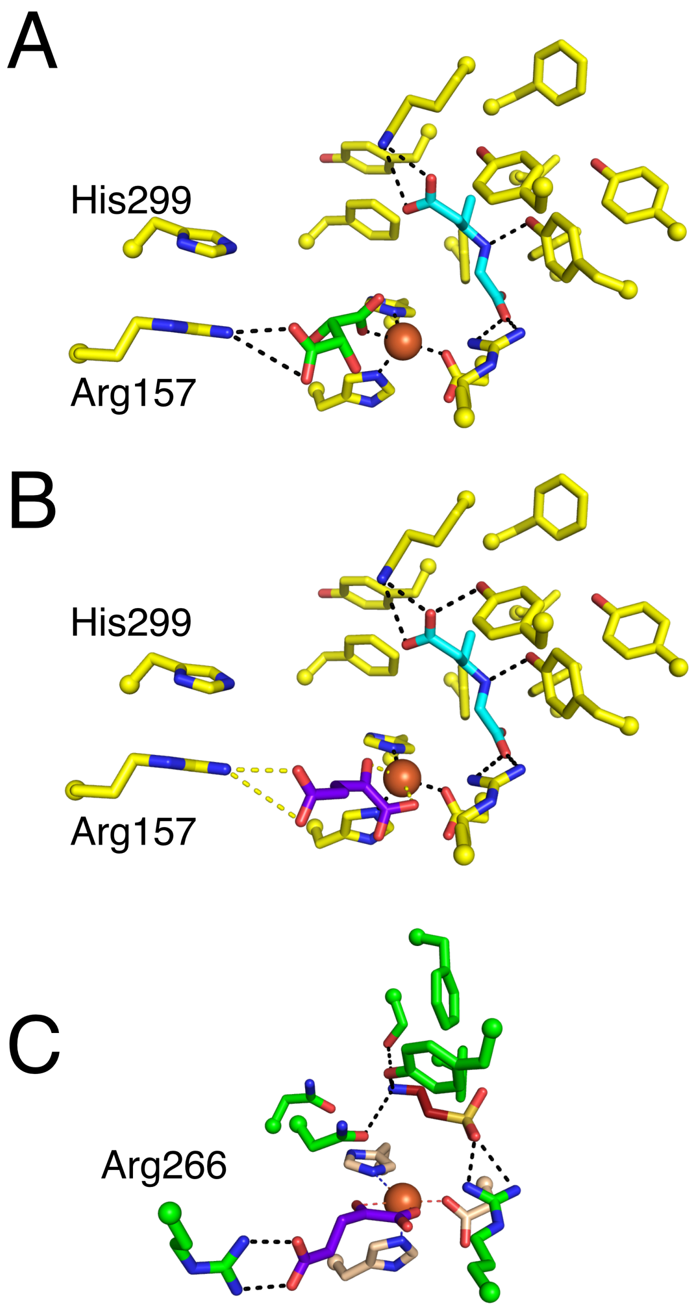

Figure S11: Active site of ScoE with tartrate bound (PDB: 6L6X) suggests an alternative binding site for α-KG. A) ScoE active site with CABA, tartrate, and Fe(II) bound (PDB: 6L6X). CABA is shown in cyan, tartrate is shown in purple, and Fe(II) is shown as an orange sphere. As observed, tartrate does not bind Fe(II) in a bidentate fashion but does not intrude on the CABA binding site. Tartrate binding is stabilized by an interaction with Arg157, which is in turn maintained in the observed conformation by a stacking interaction with His299. **B)** ScoE active site (PDB: 6L6X) with α-KG modeled in place of tartrate. CABA is shown in cyan. Modeled α-KG is shown in purple, yellow dashed lines indicate potential close interactions (less than 4.0 Å). As modeled, α-KG is 4.8 Å away from His299. Fe(II) is shown as an orange sphere. **C)** Active site of TauD (PDB: 1OS7) with α-KG shown in purple, taurine shown in red, and Fe(II) shown as an orange sphere. The facial triad is shown in wheat. Dashed lines are close interactions (less than 4.0 Å).

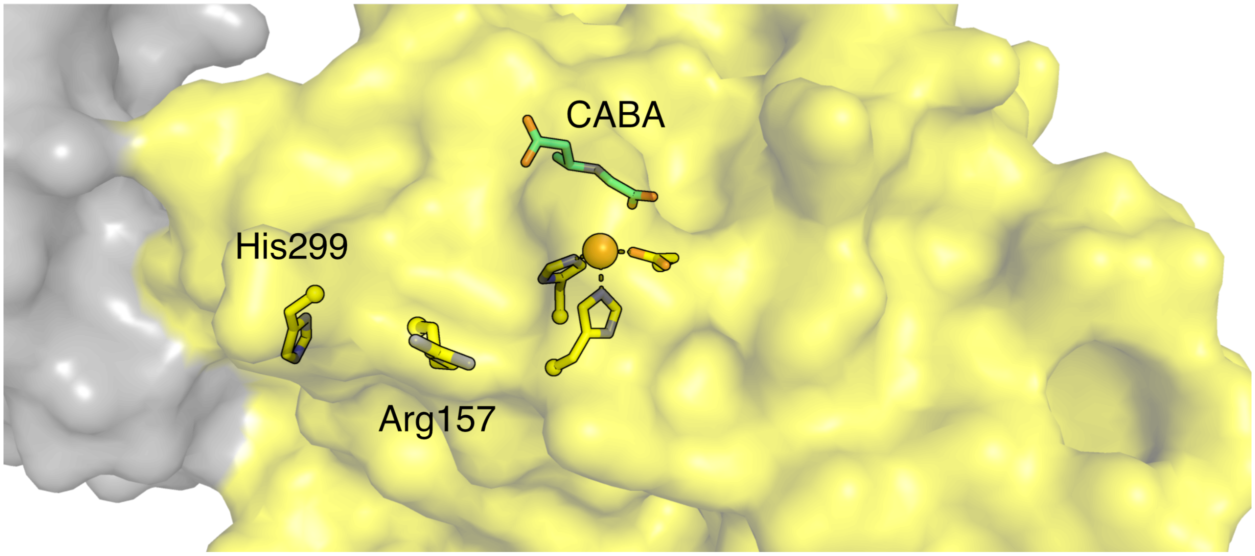

Figure S12: His299 makes lattice contacts in ScoE crystal forms presented here. CABA is indicated and Fe(II) is shown as an orange sphere. Arg157 and His299 are both shown as sticks. Arg157 is at the protein surface and His299 is at the interface with a symmetry mate, shown in gray.

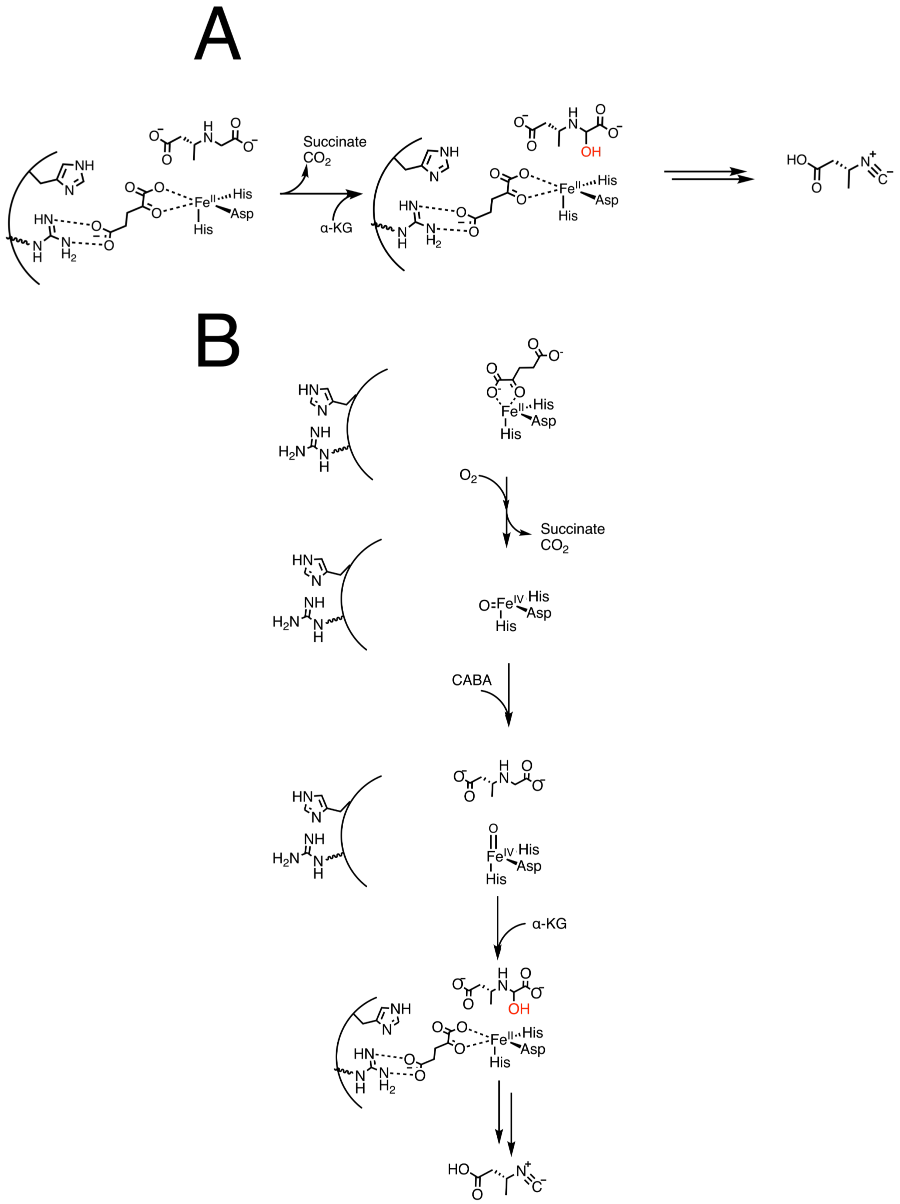

Figure S13: One α-KG binding site versus two different α-KG sites. A) α-KG may catalyze both half-reactions that lead to INBA formation by binding to a single binding site, which is comprised of His299 and Arg157 (only guanidinium moiety shown for clarity). This site has not been visualized crystallographically with α-KG bound, presumably due to either the presence of tartrate in the crystallization buffer or lattice constraints. With His299 and Arg157 flipped in toward the active site, α-KG can bind in this site without impinging on the substrate or putative intermediate. **B)** Alternatively, α-KG may utilize two binding sites. In the absence of CABA, α-KG could bind in the off-site orientation and may react with oxygen to form a protected off-line Fe(IV)=O species. CABA binding would result in the re-orientation of this Fe(IV)=O species and in CABA hydroxylation (shown as a C5 hydroxylation, although the location is unknown). With a hydroxylated CABA intermediate bound, the second α-KG would enter the active site, inducing a conformational change in His299 and Arg157, and creating a second α-KG binding site.

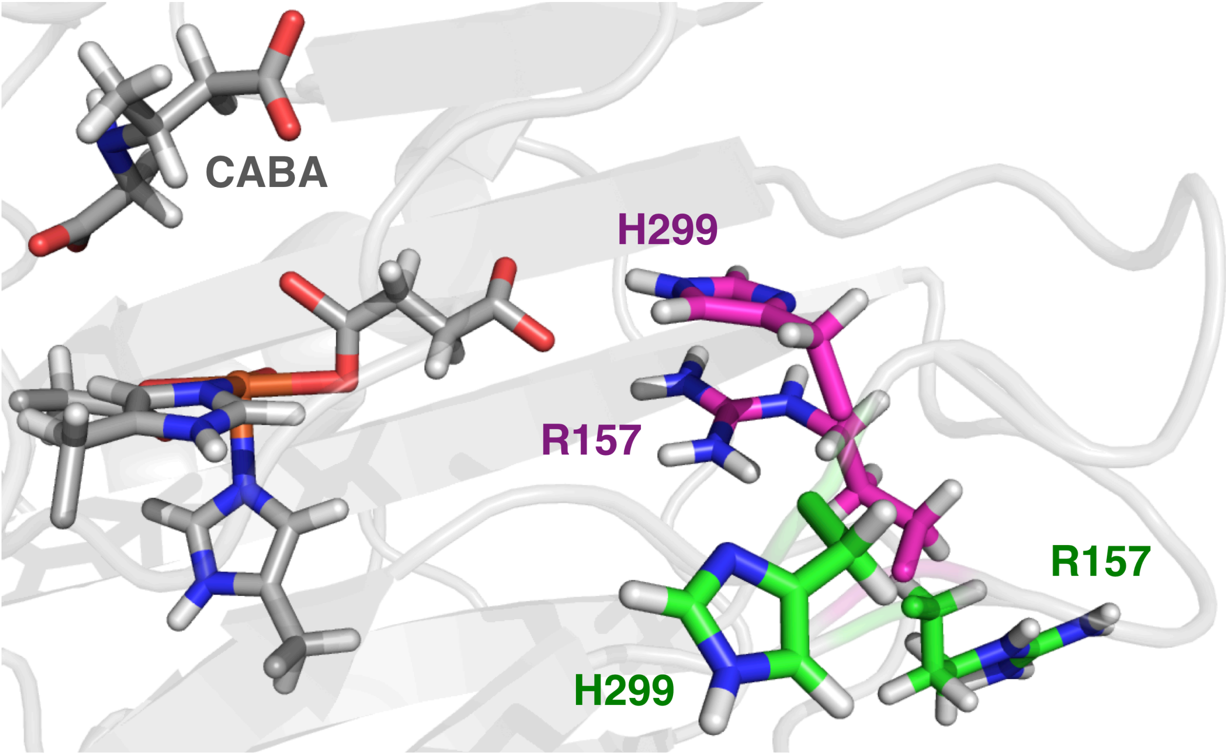

Figure S14: Aligned structures used in MD simulations. The aligned structures for CS1 and CS3 (see Methods) are shown as gray translucent cartoon and the active site and CABA are shown sticks with gray carbon atoms. The residues Arg157 and His299 in outward state are shown as sticks with green carbon atoms from CS1. The residues Arg157 and His299 in inward state are shown as sticks with green carbon atoms from CS3. Only the side chain atoms are shown for protein residues.

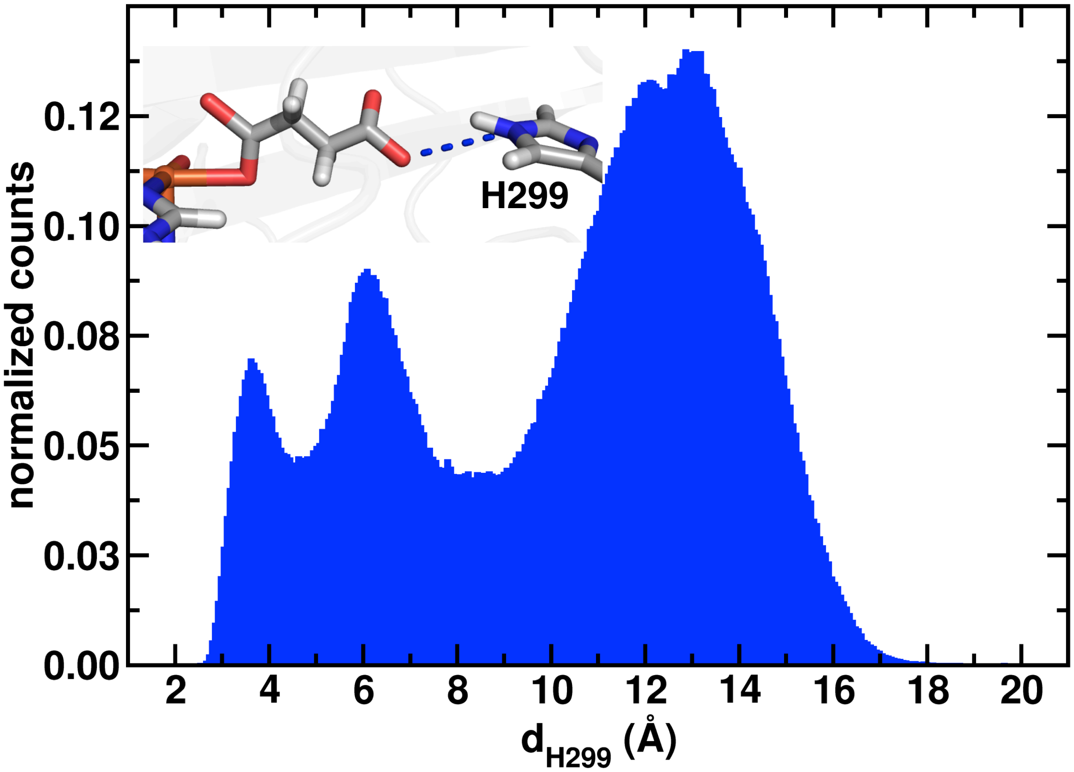

Figure S15: Distance distribution for succinate and His299 from MD simulations. The distribution for the minimum distance between succinate and His299, d_{H299}, from a total of 3.0 microseconds of dynamics with 12 different starting conformations is shown as a blue histogram.

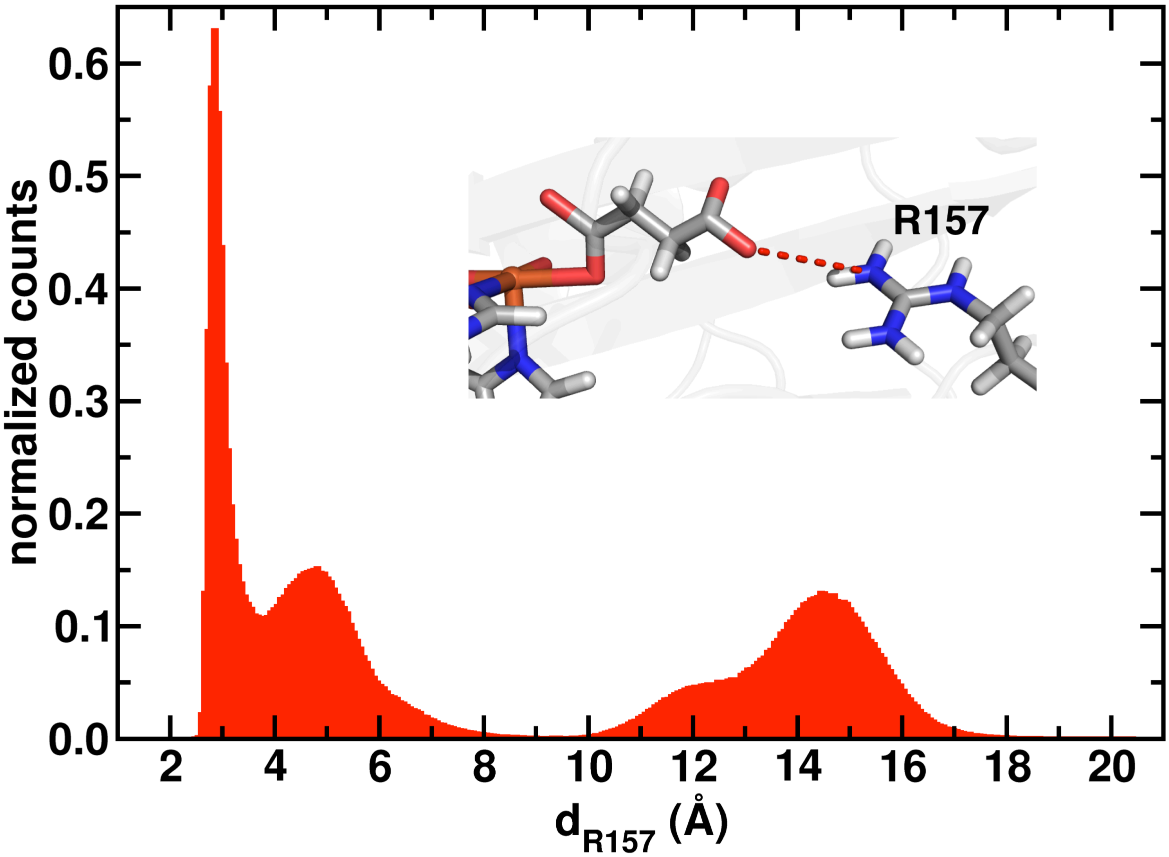

Figure S16: Distance distribution for succinate and Arg157 from MD simulations. The distribution for the minimum distance between succinate and Arg157, d_{R157}, from a total of 3.0 microseconds of dynamics with 12 different starting conformations is shown as a red histogram.

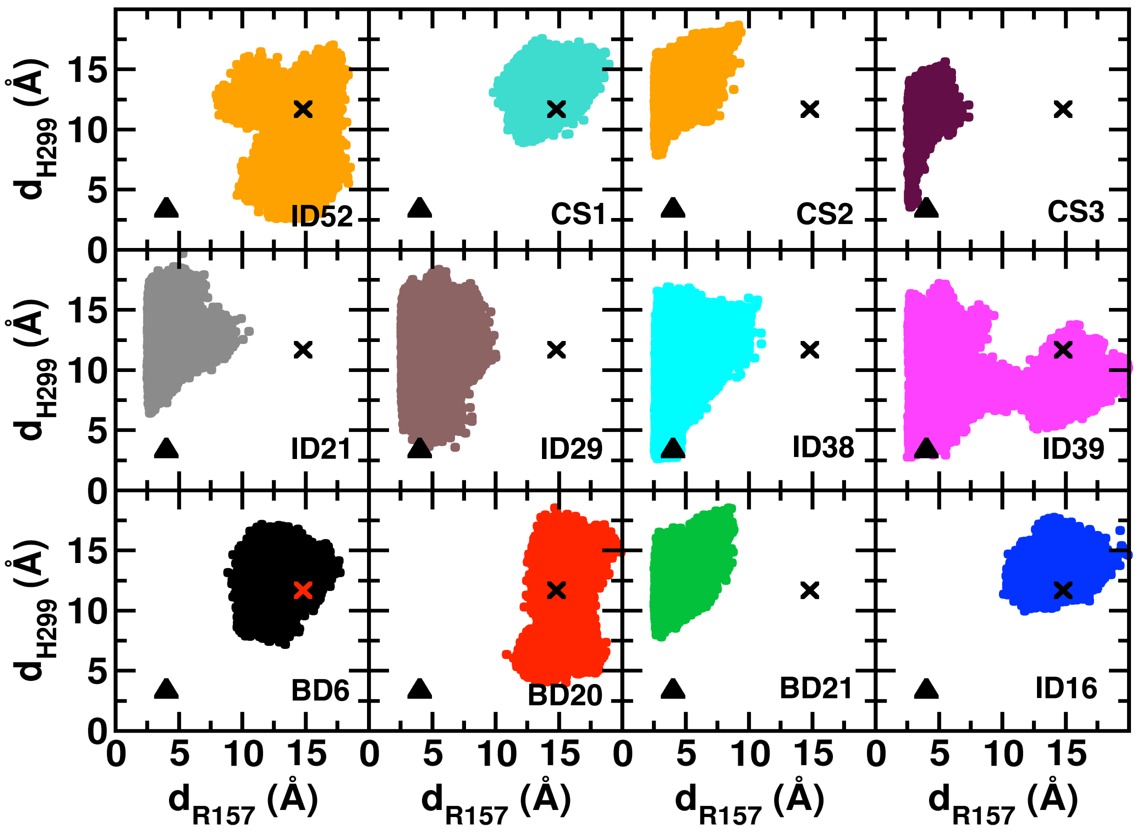

Figure S17: The distribution for the minimum distance between succinate and His299 (d_{H299}) and succinate and Arg157 (d_{R157}) in Å from 250 ns of dynamics per rotamer. The CS1 crystal structure (outward) distance for the two residues to the succinate tail is indicated as a black or red X, and the CS3 inward His299/Arg157 distance is indicated as a black triangle. Several of the structures sample a wide range of distances, but ID38 and ID39 in particular sample a range of both distances consistent with interconversion between inward and outward motion.

Table M1: List of primers used.	
**Primer**	**Sequence (5’ -> 3’)**
pET30 scoE-F	AAAGGATCCGATGCAGATCGACGAACAGCC
pET30 scoE-R	TATAAGCTTTCATGCCGCCTGGATCCCGT
pET30 R157E-F	AGGTAATTCCGGAGAAGAACGAGGGCACGTACTTCATCGACA
pET30 R157E-R	TGTCGATGAAGTACGTGCCCTCGTTCTTCTCCGGAATTACCT
pET30 R157Q-F	AGGTAATTCCGGAGAAGAACCAGGGCACGTACTTCATCGACA
pET30 R157Q-R	TGTCGATGAAGTACGTGCCCTGGTTCTTCTCCGGAATTACCT
pET30 H299Q-F	TCCACCGCGCCCGGCAGACGACCACACCCGAGCC
pET30 H299Q-R	GGCTCGGGTGTGGTCGTCTGCCGGGCGCGGTGGA
pET30 Y101-F	CCTACTACGAGCCGATGTTCCAGCACCCGGAGGTCA
pET30 Y101-R	TGACCTCCGGGTGCTGGAACATCGGCTCGTAGTAGG
pET30 Y96F-F	GGCCGCCCGGAGGCCTTCTACGAGCCGATGTACCAGCA
pET30 Y96F-R	TGCTGGTACATCGGCTCGTAGAAGGCCTCCGGGCGGCC
pET30 Y97F-F	GGCCGCCCGGAGGCCTACTTCGAGCCGATGTACCAGCA
pET30 Y97-R	TGCTGGTACATCGGCTCGAAGTAGGCCTCCGGGCGGCC
pET30 R195Q-F	GCAAGTACTTCAAGATCCAGCCCCACGATGTCTACCG
pET30 R195Q-R	CGGTAGACATCGTGGGGCTGGATCTTGAAGTACTTGC
pETCDFDuet-1 scoA-F	AAAGAATTCGATGTCACCGCATGACGACGC
pETCDFDuet-1 scoA-R	TATAAGCTTCTACTTGGCGGGCATTGCCG
pET24B scoB-F	AAACATAATGCCTGCTCCCCTCACGCT
pET24B scoB-R	AAACTCGAGTCATGCGGTGACATGGCCCG
pETCDFDuet-1 scoC-F	AAAGGATCCGATGGACCGGCTCCACCACCC
pETCDFDuet-1 scoC-R	AAACTGCAGTCAGTTGACCTTGCGTGCGG

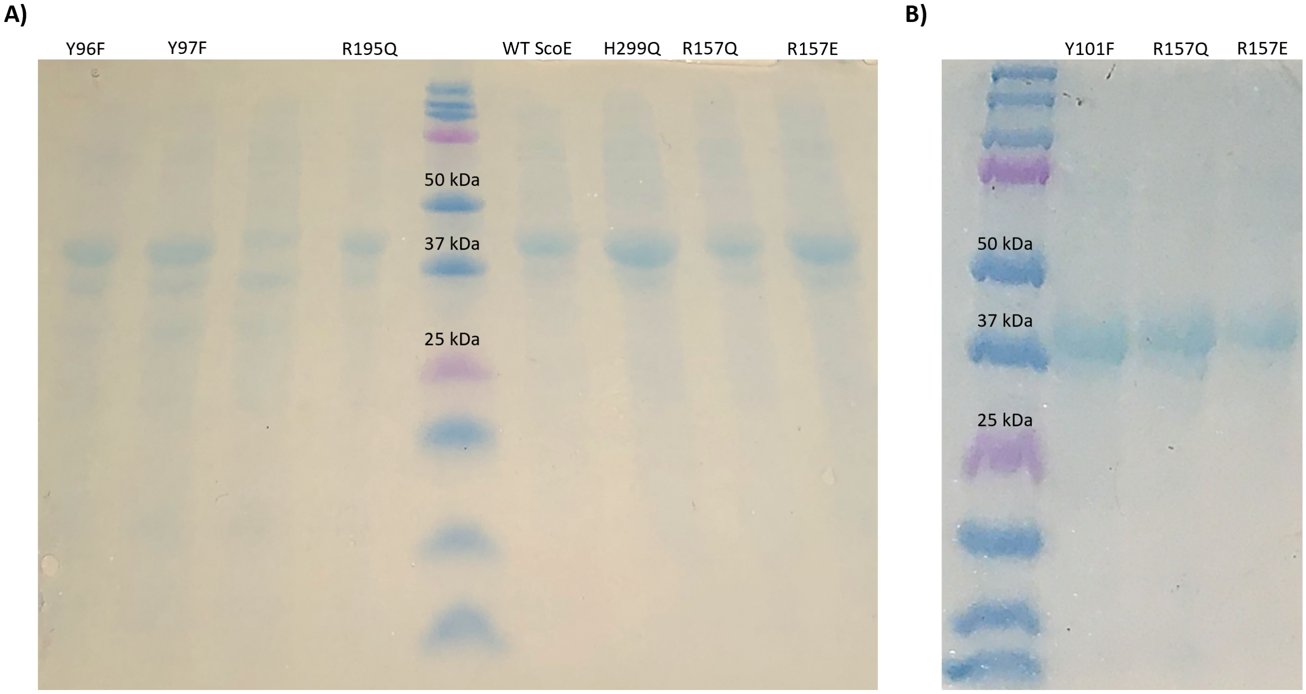

Figure M1: SDS-PAGE analysis of recombinant *E. coli* proteins purified and utilized in this study. A) ScoE and the single amino acid variants Y96F, Y97F, R195Q, H299Q, R157Q, and R157E used for *in vitro* biochemical assays. **B)** Single amino acid variants Y101F, R157Q, and R157E used for *in vitro* biochemical assays. Any kD Mini-PROTEAN TGX gels (precast, Biorad) were used for analysis of single amino acid variants of ScoE in this study. All recombinant proteins contain an N-hexahistidine tag and were largely soluble in *E. coli*.

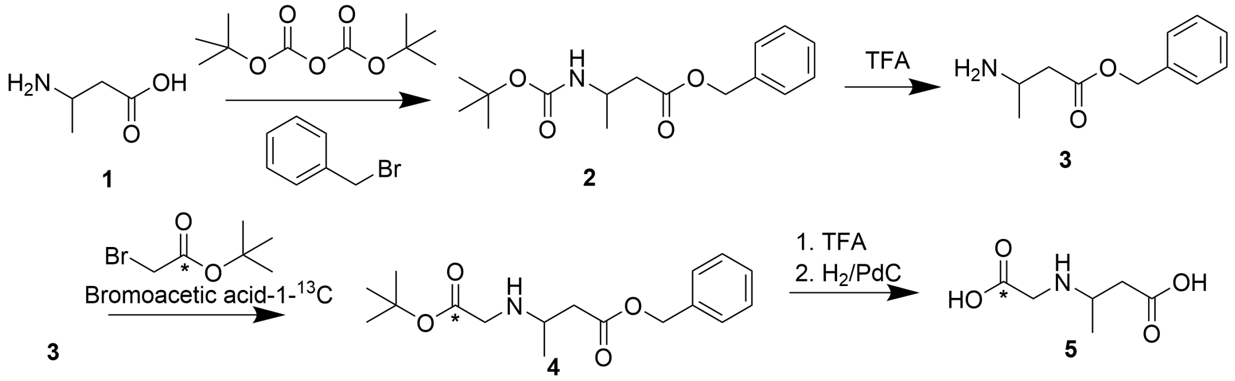

Figure M2: Synthesis of isotope labeled CABA. Compound 3 was mixed with labeled bromoacetic acid to give 5.

Table S1. Data collection and model refinement statistics for crystallography.
Values in parentheses denote highest resolution bin.

	ScoE soaked with Fe(II) and CABA<br><br>CABA-Fe(II)-ScoE structure	ScoE co-crystallized with Fe(II) and α-ketoglutarate<br><br>Fe(II)-ScoE structure with off-site α-KG	ScoE soaked with Fe(II), CABA, and α-ketoglutarate<br><br>CABA-Fe(II)-ScoE structure with off-site α-KG	ScoE co-crystallized with CABA and oxovanadium<br><br>CABA-VVO-ScoE structure
**Space group**	$P4_32_12$	$P4_32_12$	$P4_32_12$	$P4_32_12$
**Unit cell (Å)**	62.0, 62.0, 167.0, 90.0°, 90.0°, 90.0°	61.4, 61.4, 167.1, 90.0°, 90.0°, 90.0°	62.0, 62.0, 168.0, 90.0°, 90.0°, 90.0°	61.6, 61.6, 167.0, 90.0°, 90.0°, 90.0°
**Resolution (Å)**	50–1.70 (1.80–1.70)	50–1.85 (1.96–1.85)	50-1.45 (1.54-1.45)	50-2.10 (2.23-2.10)
**R_{meas}**	13.0 (149.8)	10.0 (94.5)	6.2 (52.7)	15.4 (74.8)
**$CC_{1/2}$**	99.9 (72.8)	99.9 (95.3)	99.9 (87.1)	99.9 (96.8)
**$<I/\sigma>$**	15.90 (1.65)	15.93 (2.91)	20.91 (2.48)	21.51 (5.03)
**Completeness (%)**	99.9 (99.5)	99.9 (99.5)	99.8 (99.2)	99.8 (99.1)
**Unique reflections**	36859 (5822)	28221 (4427)	59316 (9339)	19750 (3077)
**Total reflections**	474901 (72730)	357160 (56828)	612056 (53314)	450292 (65651)
**Redundancy**	12.9 (12.5)	12.7 (7.5)	10.3 (5.7)	22.7 (21.3)
**R_{work}/R_{free}**	0.166/0.196	0.169/0.207	0.169/0.192	0.171/0.213
**RMSD bond length (Å)**	0.007	0.003	0.006	0.003
**RMSD bond angles (°)**	0.86	0.635	0.863	0.630
**Chains in asymmetric unit**	1	1	1	1
**Number of:**				
**Total atoms**	2595	2555	2691	2503
**Protein atoms**	2398	2376	2402	2341
**Water molecules**	171	167	259	143
**Fe(II)**	1	1	1	N/A
**VVO**	N/A	N/A	N/A	1
**CABA**	1 molecule	0	1 molecule	1 molecule
**α-ketoglutarate**	0	1 off- site molecule	1 off-site molecule	0

**acetate**	3 molecules		1 molecule	1 molecule
**chloride**	0	1	0	2
**Ramachandran analysis**				
**Favored (%)**	98.30	98.30	98.64	98.64
**Allowed (%)**	1.70	1.70	1.36	1.36
**Disallowed (%)**	0.0	0.0	0.0	0.0
**Rotamer outliers (%)**	0.40	0.0	0.40	0.0
**Average B factors**				
**Protein (Å^2)**	22.6	26.81	18.89	29.69
**Water (Å^2)**	27.2	31.76	26.77	34.22
**CABA (Å^2)**	19.98	N/A	21.04	30.53
**Fe(II) (Å^2)**	17.0	19.90	13.33	28.38
**VVO (Å^2)**	N/A	N/A	N/A	28.38
**α-ketoglutarate (Å^2)**	N/A	27.90	18.70	N/A
**acetate (Å^2)**	37.84	N/A	41.55	48.16
**chloride (Å^2)**	41.33	32.66	N/A	56.14
**Partial Occupancies**				
**CABA (%)**	N/A	N/A	70	N/A
**α-ketoglutarate (%)**	N/A	61	30	N/A

Table S2. Minimum distance between succinate and Arg157 and His299 in MD initial configurations generated using the Pymol mutagenesis tool along with total production simulation time. Using the rotamer library in Pymol's mutagenesis tool, we generated 9 additional initial configurations of Arg157 for classical MD: 6 were taken with independence (ID) to the backbone structure, whereas 3 were chosen with backbone dependence (BD). We also report the strain induced on the X-ray structure protein for each of these Arg157 rotamer choices. The relevant distances for CS1, CS2 and CS3 are also shown for comparison.

**Rotamer**	**d_{R157} (Å)**	**d_{H299} (Å)**	**strain**	**time**
BD6	10.4	11.7	70.75	250
BD20	8.5	11.7	61.65	250
BD21	14.8	11.7	54.25	250
ID16	10.4	11.7	44.86	250
ID21	7.3	11.7	28.46	250
ID29	7.2	11.7	37.63	500
ID38	7.5	11.7	45.54	500
ID39	5.9	11.7	27.44	250
ID52	5.7	11.7	29.50	250
CS1	14.8	11.7	--	250
CS2	4.0	11.7	--	250
CS3	4.0	3.3	--	250
